# Three component synthesis of triazolo[1,2-a]indazole-trione and spiro triazolo[1,2-a]indazole-tetraones using GO/SiO_2_/Co (II)

**DOI:** 10.1038/s41598-022-22304-y

**Published:** 2022-10-25

**Authors:** Mahnaz Mirheidari, Javad Safaei-Ghomi

**Affiliations:** Department of Organic Chemistry, Faculty of Chemistry, University of Kashan, Kashan, Iran

**Keywords:** Green chemistry, Organic chemistry, Heterogeneous catalysis

## Abstract

In this study, a functionalized graphene oxide catalyst (GO/f-SiO_2_/Co) was successfully synthesized by decorating the graphene oxide surface using the attachment of hybrid silane (silica/nitrogen) and chelation with Co (II). The catalyst has been characterized by Fourier Transform Infrared (FT-IR), powder X-ray diffraction (XRD), Energy Dispersive X-ray (EDX), Scanning Electron Microscopy (SEM), Transmission electron microscopy (TEM), Raman spectra, Brunauer–Emmett–Teller (BET), and Thermal Gravimetric (TGA) analyses. The synthesized catalyst was used as an effective heterogeneous catalyst for the synthesis of triazolo[1,2-a]indazole-trione and spiro triazolo[1,2-a]indazole-tetraones derivatives under solvent-free conditions at 90 °C. The high thermal stability, corrosion resistance, and ability of the catalyst to recycle make the catalyst favorable. In addition, easy work-up procedure and short reaction time with high conversion yields (91–97%) are some benefits of the current method.

## Introduction

**Scheme 1:**
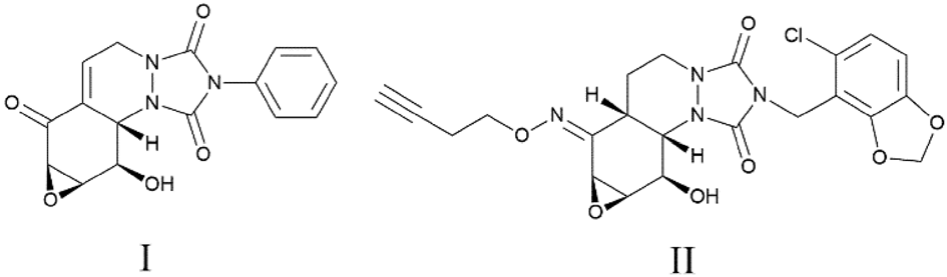
The active biological urazole compounds.

The heterocyclic compounds containing nitrogen are essential part of organic chemistry and have drawn the attention of many chemistry researchers. The numerous nitrogen compounds are widely present in nature and consider as significant molecules with biological activities^1–4^. The heterocyclic molecules with urazole moiety such as triazolo[1,2-a]indazoletrione and spirotriazolo[1,2-a]indazoletetraone have been widely found in natural and unnatural compounds like HSP-72 induction inhibitors (I,II) that they have known as alkaloids (Scheme 1). The compounds also indicate therapeutic properties such as anticancer, anticonvulsant, and hypolipidemic^5–8^. Since they are biologically active compounds; research workers have used different strategies to prepare different triazolo[1,2-a]indazoletrione and spirotriazolo[1,2-a]indazoletetraone derivatives, although they have confronted different problems such as harsh conditions, low yields, and long reaction times^9–14^.

On the other hand, researchers are trying to design new environmentally friendly heterogeneous catalysts with longer lifetimes. Graphene as an allotrope of carbon with a two-dimensional structure has received worldwide attention. It was first discovered in 2004 by Geim and Novoselov^15^. The graphene with outstanding physical, chemical and mechanical features and environmentally friendly character, has become an influential part in chemistry^16, 17^. The Graphene oxide is an oxidized form of the graphene which is made via the oxidation of graphite crystals and it is including various oxygen groups like hydroxyl, epoxides, and carboxyl groups acting as the active catalytic sites. The functionalization of these groups imparts remarkable properties to graphene oxide^18–20^ and produces graphene-based hybrid materials with various applications^21–23^.It has been of interest for its easy preparation, superb activity, water solubility, and low-cost production^24, 25^.

Silica, as a non-metallic material, has properties like corrosion resistance, high chemical and thermal stability, and facile dispersion in solvents^26, 27^. Also, it is an inexpensive and non-toxic material. SiO_2_ has many hydroxyl groups on its surface that supply surface modification capability for silica. The silica nanoparticles are unstable and could be agglomerated and lose nanoscale dimension and activity. To solve the problem, we stabilize the functionalized silica nanoparticles on the graphene oxide with various oxygen groups like hydroxyl, epoxides, and carboxyl. Graphene oxide and silica have interaction with each other to increase the catalytic effect. The attachment could improve the homogenous dispersion, water resistance, interfacial tension properties, and application of graphene oxide^28, 29^.

Also, the modification of the treatment of graphene oxide with silica through covalent bonds enhances corrosion resistance, hydrophilicity properties, and thermal resistance of graphene oxide^30–32^.

The presence of metal nanoparticles on graphene sheets would lead to more acceptable activity and stability of the materials for various applications^33, 34^. Due to the high cost of noble metals, cheaper metals such as Fe, Ni, and Co could be replaced for this purpose^35^.

**Scheme 2:**
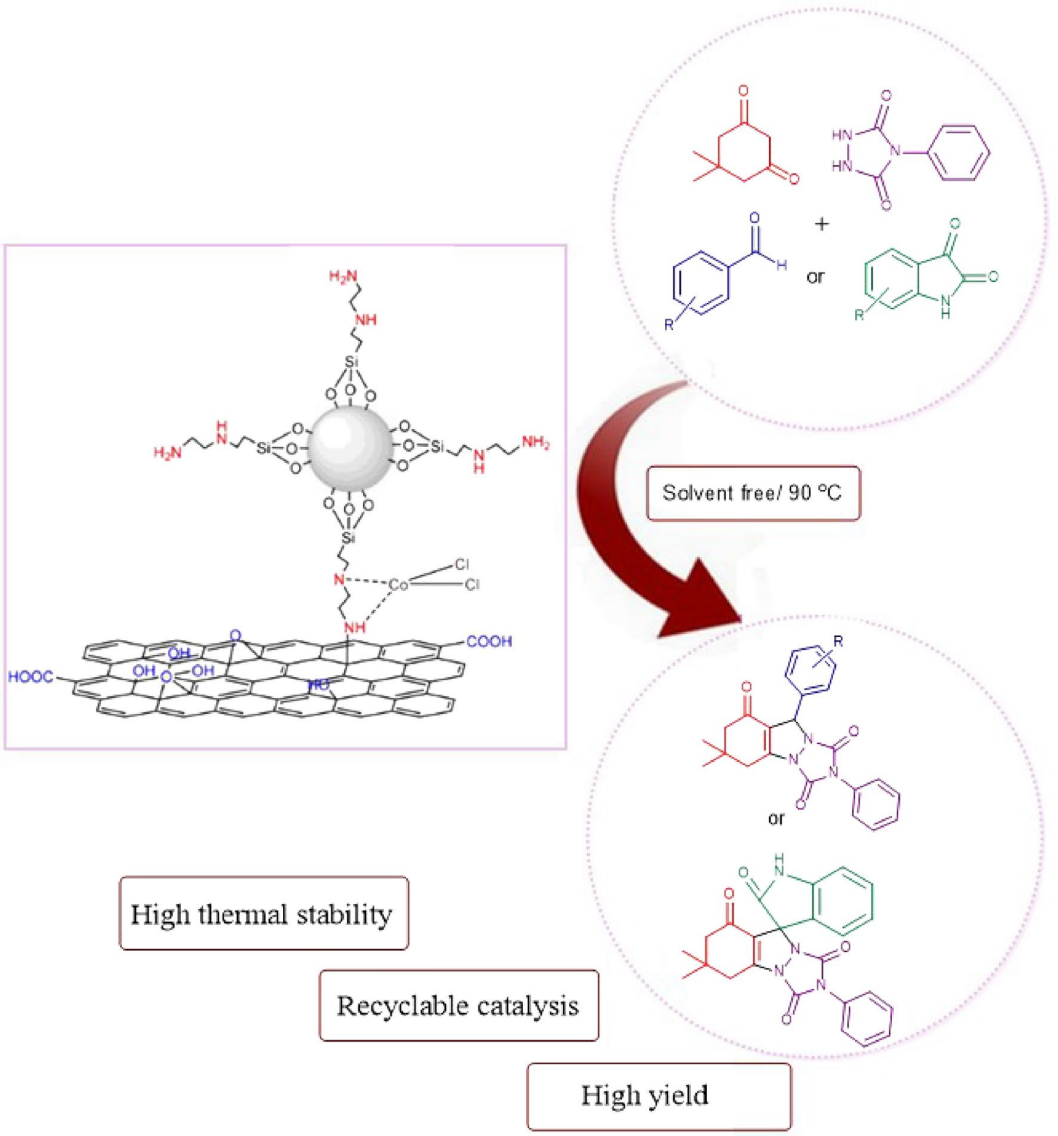
The use of GO/SiO_2_/Co (II) in the synthesis of triazolo[1,2-a]indazole-trione and spiro triazolo[1,2-a]indazole-tetraones.

The above fact promoted us to find a new strategy for the high-yield synthesis of triazolo[1,2-a]indazole-trione and spiro triazolo[1,2-a]indazole-tetraones in the presence of modified graphene oxide (GO/f–SiO_2_/Co) as a heterogeneous catalyst (Scheme 2).

## Experimental section

**Scheme 3:**
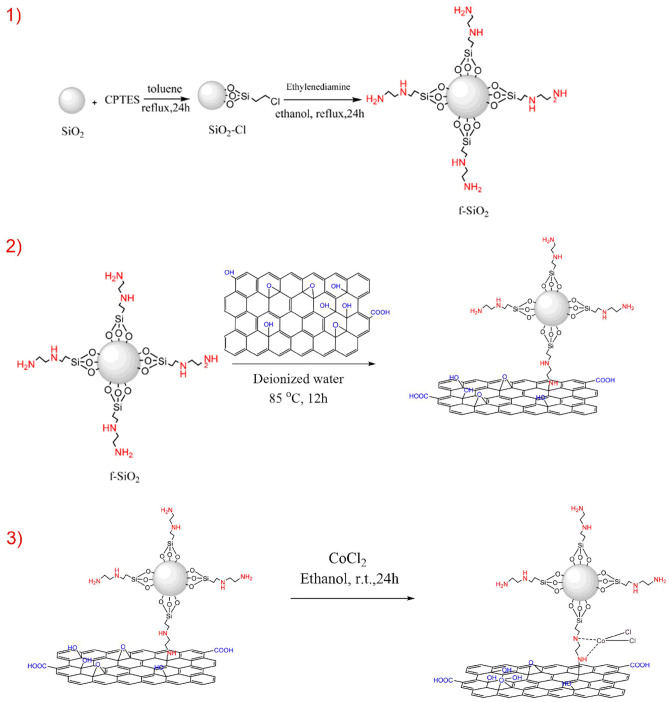
The synthesis of the catalyst (GO/f-SiO_2_/Co).

To prepare the catalyst, the graphene oxide was first synthesized. In the next stage, ethylenediamine as a linker under reflux conditions attached to silica with the high surface area through covalent bonds (GO/f–SiO_2_). Then, Co (II) chelated to GO/f-SiO_2_ via an easy process to make an efficient catalyst (Scheme 3).

**Figure 1:**
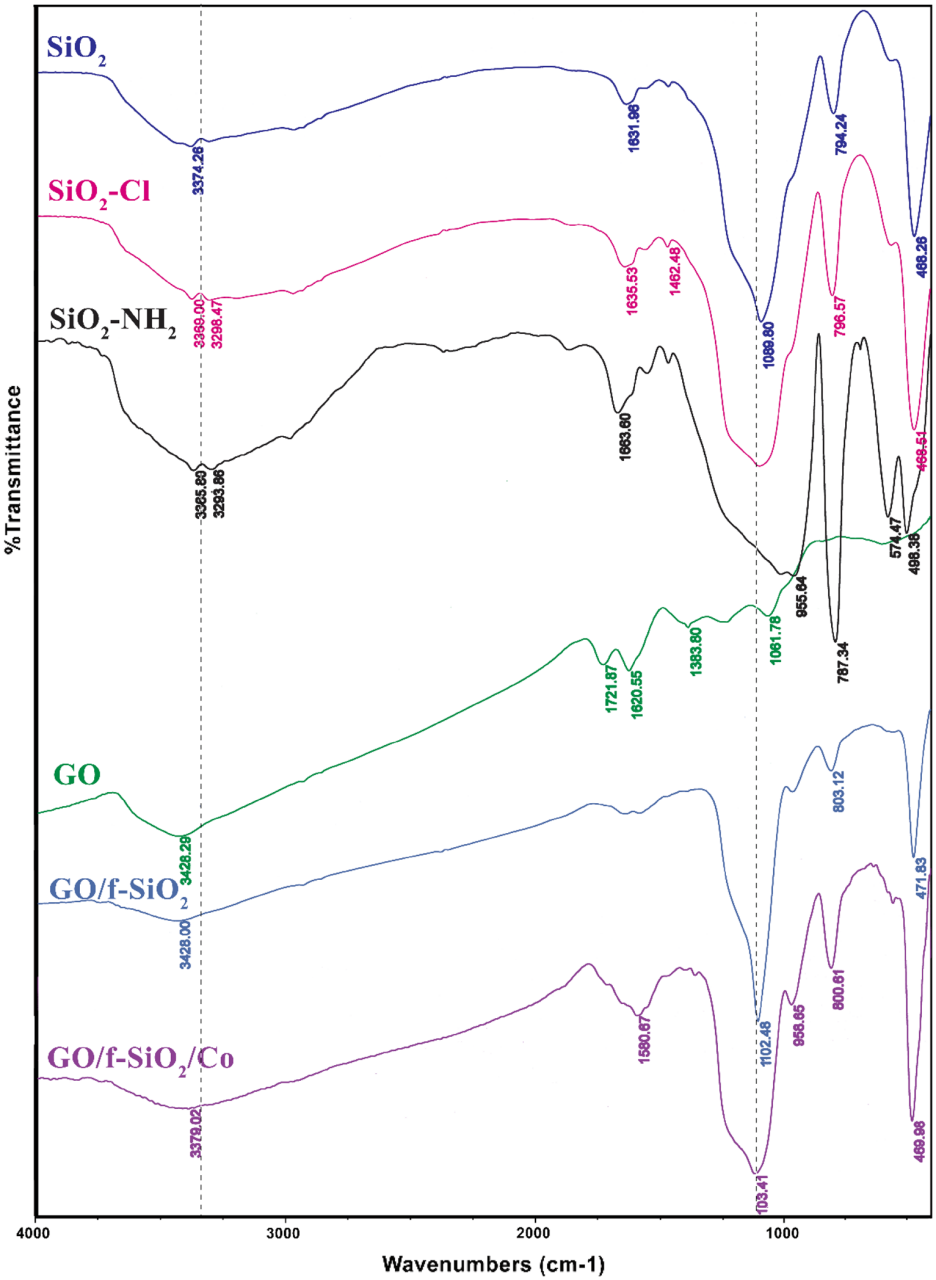
IR spectra for different synthesis parts of the catalyst.

The FT-IR spectra for the catalyst are given in Fig. 1. The Graphene oxide shows a peak at 3398 cm^−1^ which is attributed to stretching vibrations of O–H group. The characteristic band with low intensity at 1730 cm^−1^ describes carbonyl groups vibrations which are in the edges of the graphene oxide. The C-O band for the epoxy group appears at 1383 cm^−1^. The spectrum for silica exhibits a high-intensity absorption peak at 1089 cm^−1^ corresponding to asymmetric vibrations of the Si–O–Si bonds. The band at 3370 cm^-1^ also describes hydroxyl group vibrations. SiO_2_-Cl and SiO_2_-ethylenediamine produce new bands at around 2950 cm^-1^ related to ethoxy moieties vibrations. The presence of characteristic peaks in the final catalyst spectrum relating to the graphene oxide and organic–inorganic hybrid parts confirm grafting the f-SiO_2_ part on the surface of the graphene oxide.

**Figure 2:**
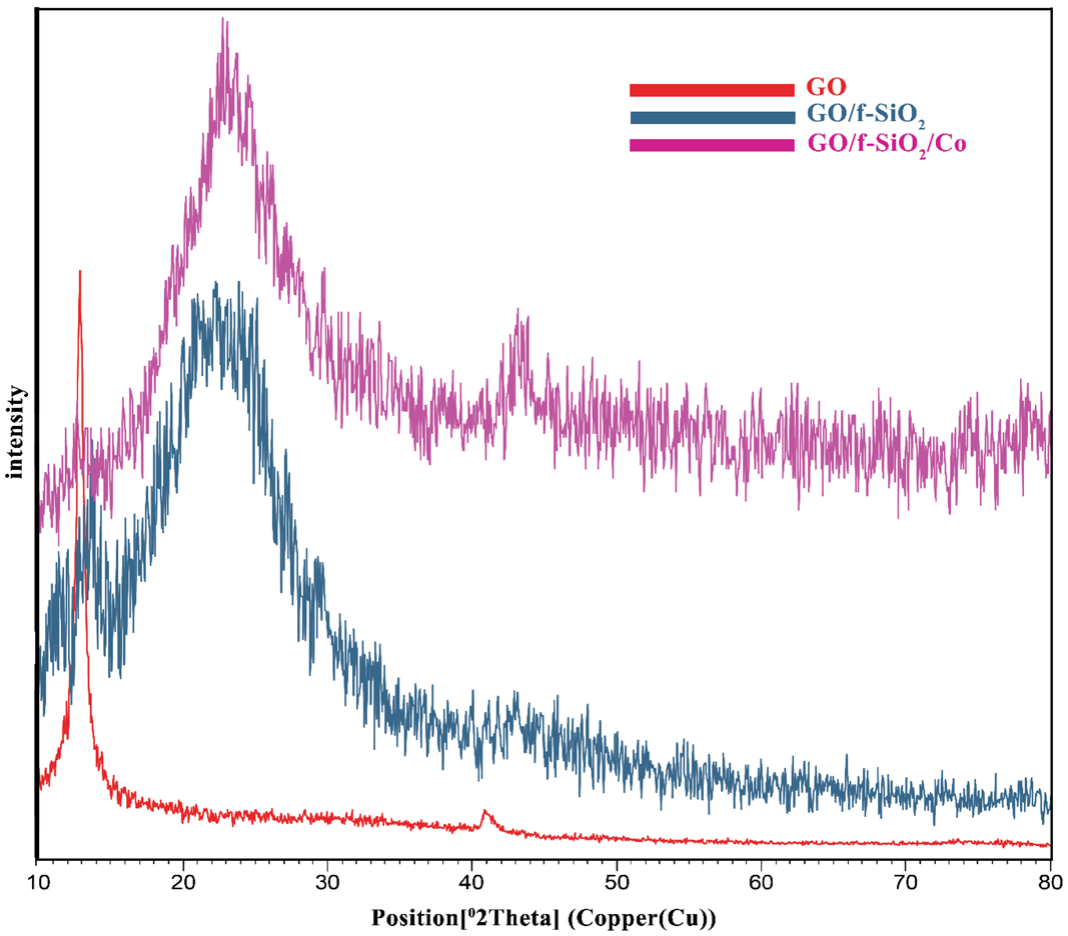
XRD patterns for GO, GO/f-SiO_2_, GO/f-SiO_2_/Co.

XRD patterns for the catalyst in different steps are depicted in Fig. 2. The sheets of graphene oxide indicate a strong characteristic peak at 2θ = 13° with d-spacing of 0.08 nm that is attributed to the (001) plane. The large amount of a d-spacing is attributed to the formation of oxygen functional groups on the plane of graphite, which makes spaces between layers^36^. In the XRD patterns of GO/f–SiO_2_ and GO/f-SiO_2_/Co, the peaks at 2θ = 25° and 2θ = 44° are related to amorphous SiO_2_ and cobalt, respectively. The diffraction patterns show decreases in the peak intensity belonging to the graphene oxide because of the removal of many oxygen-containing functional groups on the surface of graphene oxide.

**Figure 3:**
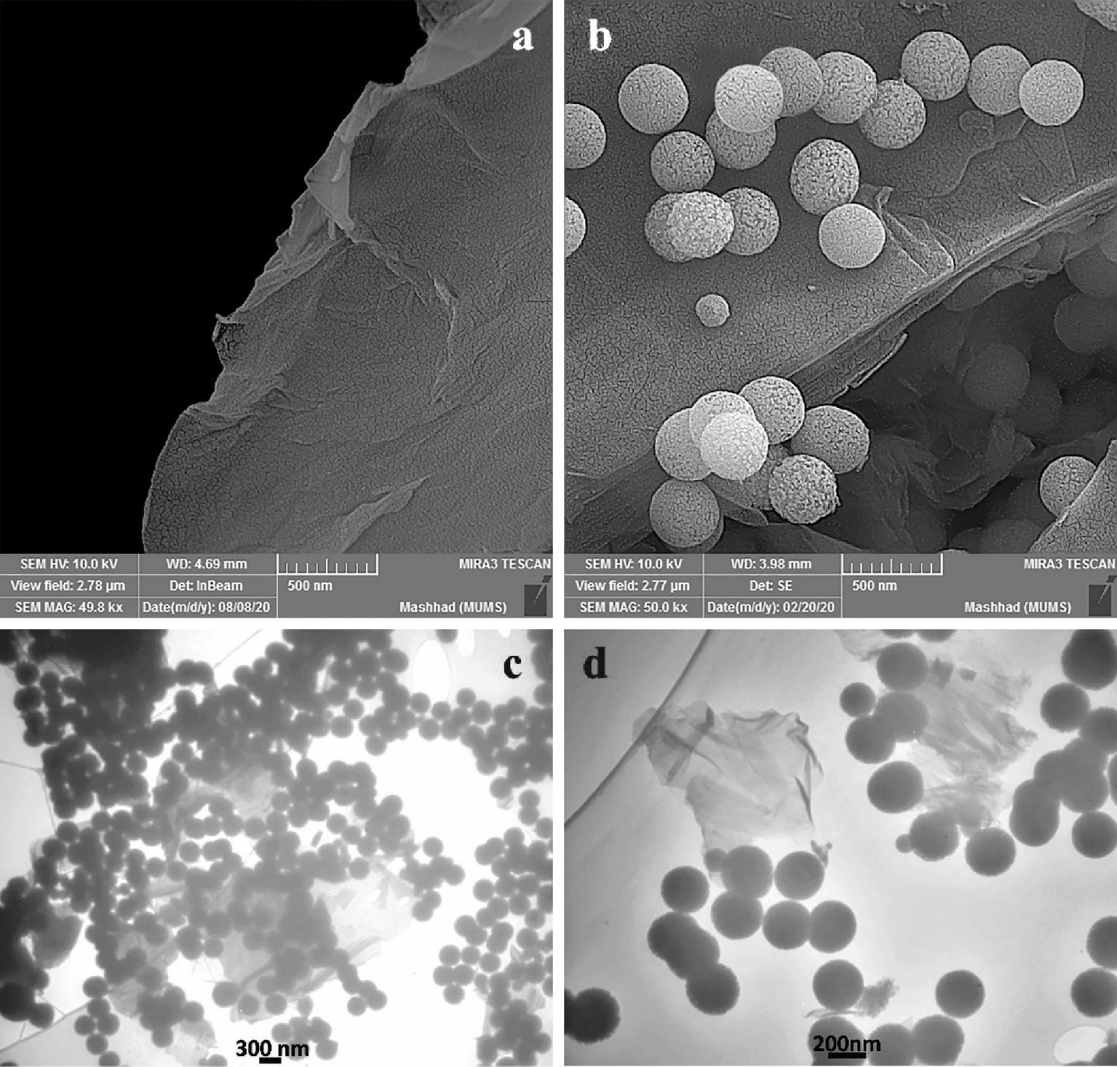
SEM images of GO (**a**), GO/f-SiO_2_/Co (**b**), TEM images of GO/f-SiO_2_/Co (**c**, **d**).

The SEM and TEM images of the catalyst are represented in Fig. 3. The SEM image for graphene oxide clearly shows the flat layer structure of graphene oxide with wrinkled sheets at the edges (Fig. 3a). The SEM image for GO/f–SiO_2_/Co plainly exhibits a uniform deposition of the modified silica with a spherical shape on the graphene oxide surface (Fig. 3b). In agreement with the SEM images, the TEM images for GO/f–SiO_2_/Co also evidently depict the presence of functionalized spherical SiO_2_ on the smooth layer of the graphene oxide. The thin and transparent layer of graphene oxide verifies a high success rate in the exfoliation of graphite into the graphene oxide (Fig. 3 c, d)^37, 38^.

Kindly check and confirm the section headings are correctly identified.The section headings are correct. 

**Figure 4:**
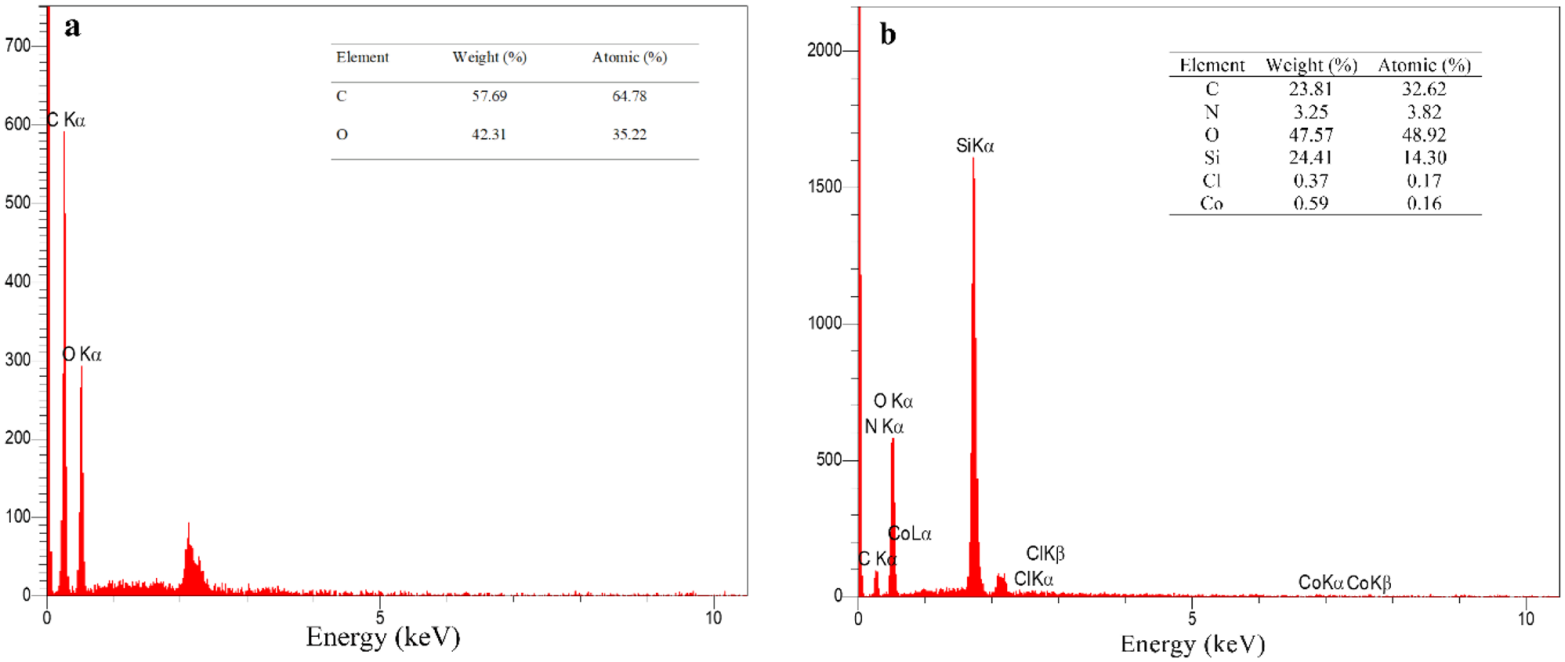
The EDX spectra of GO (**a**), GO/f-SiO_2_/Co (**b**).

The EDX analyses are shown in Fig. 4. According to the data, the ratio of carbon/oxygen in GO is roughly 2:1(Fig. 4a). In the final catalyst, the ratio of carbon/oxygen is around 1:2 (Fig. 4b). Increase in the amount of oxygen than carbon in the final catalyst is because of the presence of SiO_2_ moiety in the catalyst.

**Figure 5:**
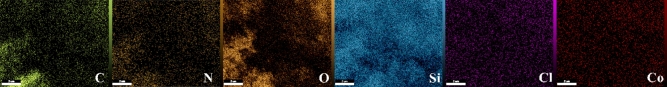
The elemental mapping of GO/f-SiO_2_/Co.

The elemental mapping images for GO/f–SiO_2_/Co also indicate the homogeneous distribution of all elements in the final catalyst (Fig. 5).

**Figure 6:**
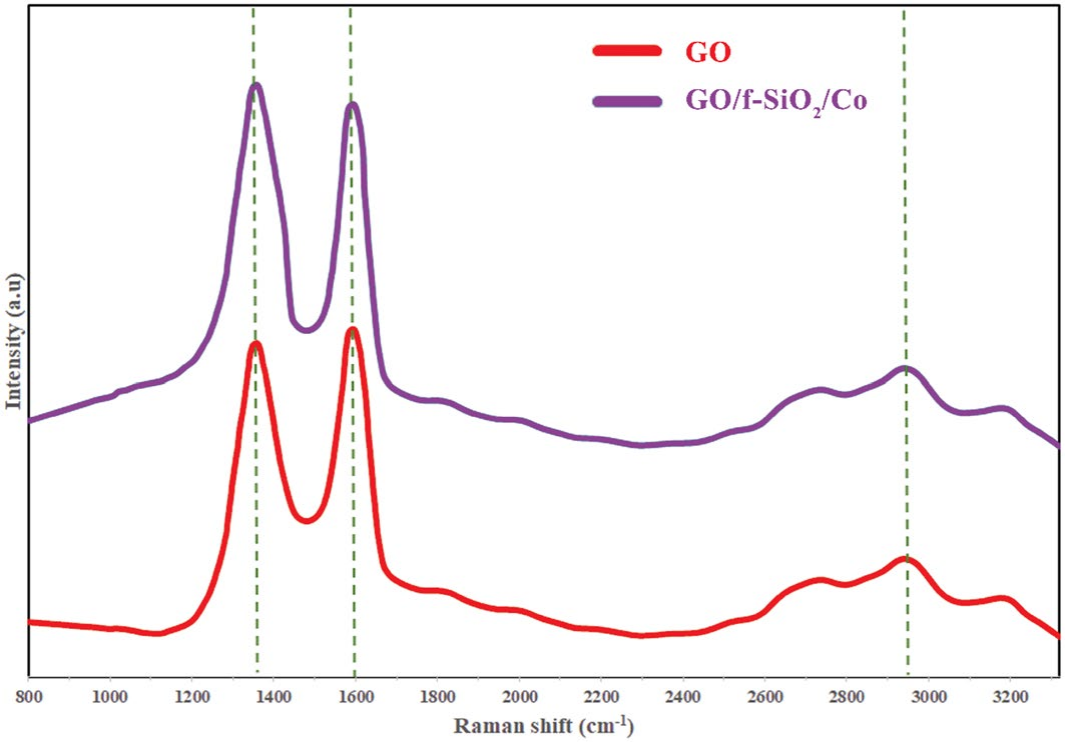
The Raman spectra of GO (**a**), GO/f-SiO_2_/Co (**b**).

The Raman spectra for GO and GO/f–SiO_2_/Co are shown in Fig. 6. The two fundamental peaks at 1362 and 1595 cm^−1^ for graphene oxide are related to D and G bands, respectively. Also, the Raman spectrum for GO/f-SiO_2_/Co depicts these peaks with a slight increase in the ratio of I_D_/I_G_ that demonstrates more transition from sp^2^ to sp^3^ due to the prosperous grafting of f-SiO_2_ on the graphene oxide^39, 40^.

**Figure 7:**
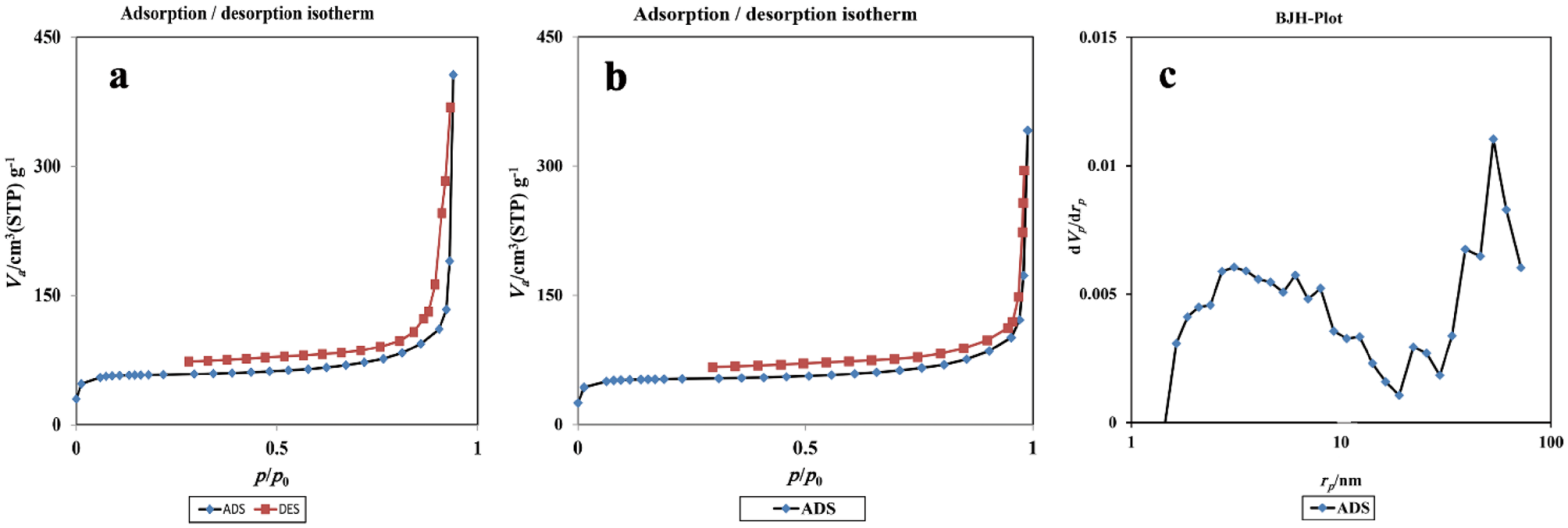
The BET for Go/f-SiO_2_ (**a**), Go/f-SiO_2_/Co (**b**), and BJH for Go/f-SiO_2_/Co (**c**).

The textural properties for GO/f-SiO_2_ and GO/f–SiO_2_/Co through N_2_ sorption measurements show high surface areas of 234.63 m^2^/g^-1^ and 205.86 m^2^/g^−1^, respectively. The narrow hysteresis loops indicate the slit-type pores between graphene oxide sheets (Fig. 7)^41^.

**Figure 8:**
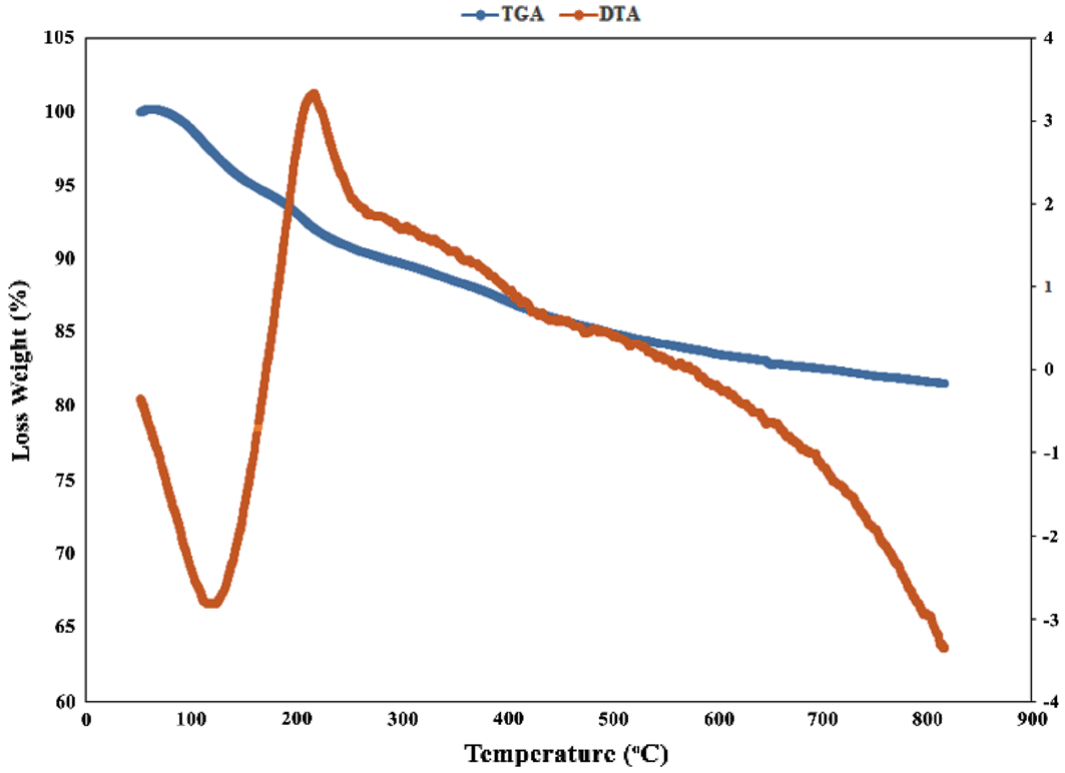
The TGA/DTA curves of GO/f-SiO_2_/Co.

According to the differential thermal analysis (DTA)/Thermogravimetric analysis (TGA) for the final catalyst (Fig. 8), the primary stage of decomposition occurs under 200 °C which is related to the evaporation of molecules like water. The main weight loss is at 220 °C and continues at a slow rate up to 800 °C with a 18% mass loss. The weight loss is related to the decomposition of organic functional groups on the graphene oxide surface^42^.

To optimize the best possible conditions to get high production yield and show the proficiency of the catalyst, various reaction conditions were examined in the model reaction (isatin, 4-phenylurazole and dimedone). The results revealed the performing of the reaction under the solvent-free conditions with the use of 20 mg of catalyst (GO/f–SiO_2_/Co) at 90 °C enhance production yield (Table 1, entry 7). As shown in Table 1, the presence of Co in the final catalyst with Lewis basic character promotes the conversion of raw materials into the final product.

**Table 1:** Optimized conditions to prepare triazolo[1,2-a]indazole-trione and spiro triazolo[1,2-a]indazole-tetraones.

Entry	Catalyst	Solvent/T (^o^C)	Catalyst (mg)	Time (min)	Yield ^a^ (%)
1	P-TSOH	Solvent-free/90 °C	20	270	57
2	CH_3_COOH	Solvent-free/90 °C	20	290	51
3	SiO_2_	Solvent-free/90 °C	20	260	56
4	GO/f–SiO_2_	Ethanol/90 °C	20	150	61
5	GO/f–SiO_2_/Co	Solvent-free /50 °C	20	90	69
6	GO/f–SiO_2_/Co	Solvent-free /70 °C	20	60	77
7	GO/f–SiO_2_/Co	Ethanol/90 °C	20	100	76
8	GO/f–SiO_2_/Co	Solvent-free /90 °C	20	19	93
9	GO/f–SiO_2_/Co	H_2_O/90 °C	20	90	79
10	GO/f–SiO_2_	Solvent-free /90 °C	20	45	81
11	GO/f–SiO_2_/Co	MeCN/90 °C	20	160	61

^a^Isolated yields.

The results related to the various triazolo[1,2-a]indazole-trione and spiro triazolo[1,2-a]indazole-tetraones derived from aryl aldehydes and isatin derivatives under solvent-free conditions at 90 °C were presented in Table 2.

**Table 2:** synthesis of triazolo[1,2-a]indazole-trione and spiro triazolo[1,2-a]indazole-tetraones ^a^.


Entry	product	Time (min)	Yield^b^(%)	M.P. /M.P. (^o^C)^c^
1		10	91	194–197/195–197^43^
2		14	96	177–178/171–173^10^
3		18	97	187–189/190–192^10^
4		11	97	131–132/131–33^43^
5		12	97	163–165/166–168^44^
6		19	93	295–298
7		12	94	230–232/298–300^12^
8		12	94	293–294/292–293^12^

^a^reaction conditions: dimedone, 4-Phenyl urazole, aryl aldehydes or isatin derivatives under solvent-free conditions, 90 °C. ^b^isolated yields. ^c^Literature references.

### Proposed mechanism

**Scheme 4:**
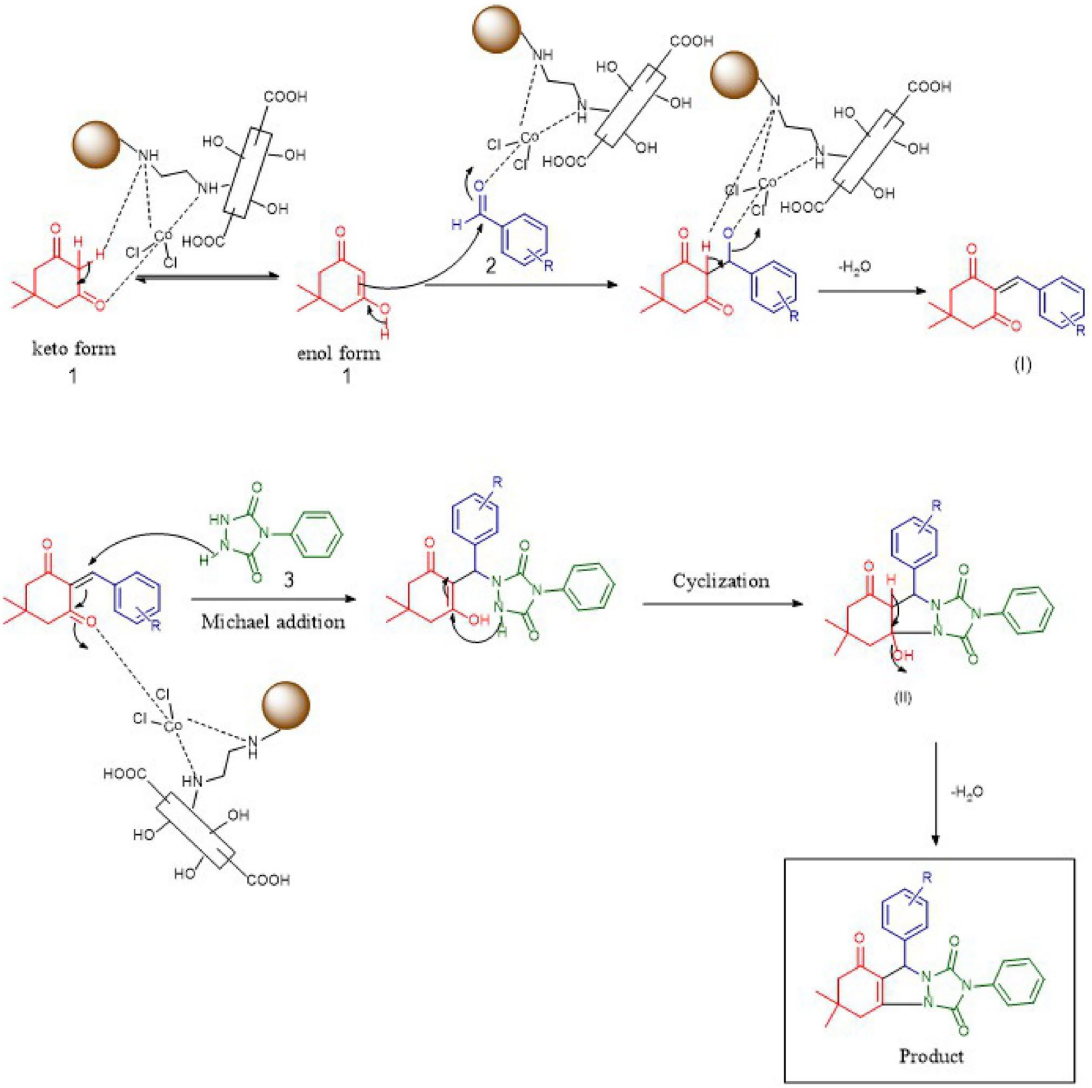
The rational mechanism for the synthesis of triazolo[1,2-a]indazole-trione and spiro triazolo[1,2-a]indazole-tetraones.

In accordance results, dimedone which has the keto-enol form (1), via an enol form reacts with benzaldehyde (2) that catalyst activates it to produce an intermediate (I). In this step, the intermediate (I) forms in a result of water removal. To form intermediate (II), the nitrogen of 4-phenyl urazole (3) reacts with the unsaturated carbon in conjugation with the carbonyl through Michel addition. In the final step, one molecule of water is removed from intermediate (II) to produce the final product (Scheme 4).

### Reusability of the catalyst

**Figure 9:**
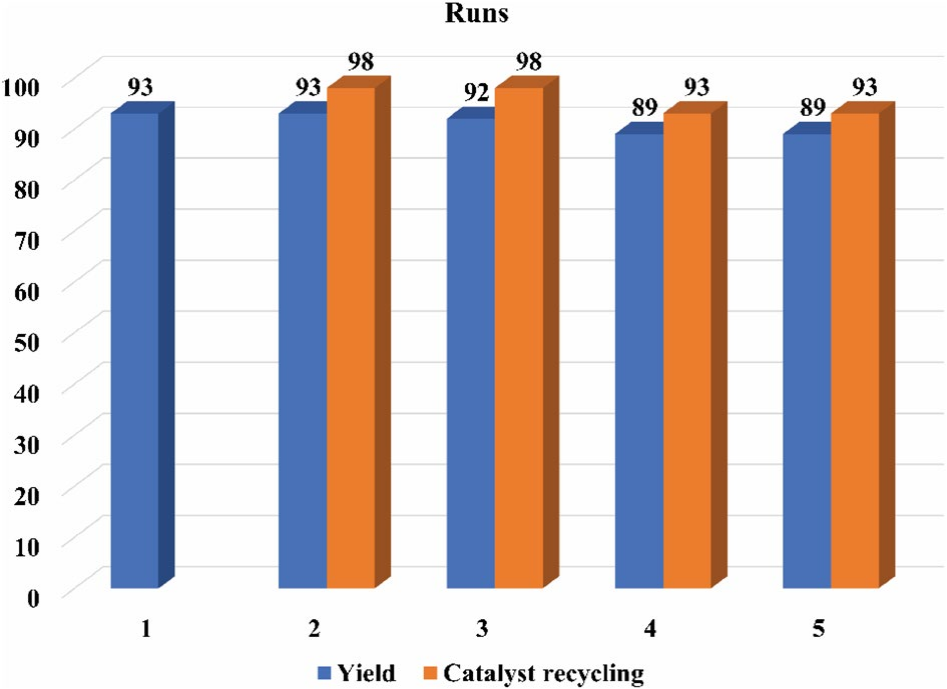
Recycling values for GO/f-SiO_2_/Co.

To estimate the catalyst reusability, after finishing the reaction process, the catalyst was discrete from the mixture and washed a few times with ethyl acetate and dried at 50 °C. As shown in Fig. 9, the catalyst was applied five times in the similar conditions with a recovering value from 98% in the second run to 93% in the fifth run. Also, this catalyst was also used five times with a slightly decline in performance.

The comparison of the results between this work and previous works for the model reaction displays our procedure in the presence of GO/f-SiO_2_/Co catalyst gives the better yield in less time (Table 3).

**Table 3:** comparison the various catalysts for the synthesis of spiro triazolo[1,2-a]indazole-tetraones.

Entry	Catalyst	Solvent	Time	Yield (%)	Ref
1	FeCl_3_	CH_3_CN	90 min	85	^8^
2	PEG-SO_3_H	Solvent-free	6 h	89	^12^
3	GO/f-SiO_2_/Co	Solvent-free	19 min	93	This work

### Substances and methods

Ethylenediamine, 3-chloropropyl triethoxysilane, Polyvinylpyrrolidone, Tetraethyl orthosilicate, dried Toluene, absolute Ethanol, dimedone, 4-Phenyl urazole, aryl aldehydes, and isatin derivatives all were purchased commercially from Sigma-Aldrich and Merck.

Melting point of products were measured by Electro thermal 9200. FT-IR measurements were carried out on a Nicolet Magna-IR 550 spectrometer using KBr plates. ^1^H NMR data were recorded in DMSO-d6 on a Bruker Avance DRX instrument. XRD patterns were obtained by an X'PertPro (Philips, PW 1510, Netherlands) instrument. Thermal analysis was carried out on a Bahr STA type.

The scanning electron microscopy (FE-SEM) and energy-dispersive X-ray analysis were carried out by (MIRA3-TESCAN FESEM). The transmission electron microscopy (TEM) image analysis was carried out using Zeiss-EM10C-100 Kv. The N_2_ adsorption/desorption was measured at 77 K using an automated gas adsorption analyzer (BELSORP-mini, Japan).

### Synthesis of catalyst

At first, graphene oxide was synthesized via modified Hummer method^45^. In continuation, 3 ml TEOS and 0.1 mmol PVP were dissolved in the solution including 70 ml mixture of water and ethanol and sonicated for 30 min. Then, 0.1 ml ethylenediamine was added dropwise within 10 min under ultrasonic. After 30 min, the produced SiO_2_ was isolated by centrifugation and washed with ethanol and water. The resulted product was dried for overnight at 80 °C. To the synthesis of functionalized graphene oxide (f–SiO_2_/Co), (0.5 ml, 5 mmol) 3-chloropropyl triethoxysilane (CPTES) was added to the stirred solution of SiO_2_ (1 g) in dry toluene (30 ml) and refluxed for 24 h. The obtained impure product was separated and washed with toluene and dried under 120 °C in a vacuum oven for 8 h to obtain the white powder as SiO_2_/CPTES. Then, Ethylenediamine (0.3 g, 1 mmol) was added to the suspension of SiO_2_/CPTES (1 g) in absolute ethanol (30 ml) and heated under reflux for 24 h. The resulting solid was collected by filtration and washed successively with ethanol and dried for overnight at 90 °C. Then, SiO_2_/ethylenediamine (f-SiO_2_) (0.16 g) was added to the container including 0.04 g GO dispersed in 20 ml of distilled water and sonicated for 20 min. The solution was stirred at 85 °C in an oil bath for 12 h. Lastly, the resulting product was collected by centrifugation, washed with deionized water and ethanol and then dried at 60 °C. In the final step, 0.1 g of GO/f–SiO_2_ with 0.01%wt CoCl_2_ was reacted in absolute ethanol for 24 h at room temperature. The final catalyst was collected and washed thoroughly with ethanol and deionized water. The product was dried at room temperature for 24 h to obtain GO/f–SiO_2_/Co catalyst.

### General procedure for the synthesis of triazolo[1,2-a]indazole-trione and spiro triazolo[1,2-a]indazole-tetraones compounds

In a 50 ml round-bottom flask, dimedone (1 mmol), 4-phenyl urazole (1 mmol), aryl aldehydes or isatin derivatives (1 mmol) and 20 mg of the catalyst (GO/f-SiO2/Co) were placed and mixed at 90 °C under solvent- free conditions until the completion of the reaction (developing of the reaction was monitored by TLC). The obtained products were washed by ethanol and then dried to get the pure compounds.

### Analysis and characterization of the synthesized compounds

The IR spectrum of the compound 4f. shows an absorption signal at 3439 cm^−1^ for the presence of NH group in the molecule structure. The absorption peaks at 1782, 1726 and 1665 cm^−1^ are assigned to the carbonyl stretching vibrations. Furthermore, the C=C stretching band is found at 1622 cm^−1^.

The ^1^HNMR analysis shows a singlet signal at δ = 10.93 ppm for NH proton. The signals at the reign of δ = 7.53–6.89 ppm is arising from hydrogens of aromatic moieties. Hydrogens of 2CH_2_ in dimedone appear as two doublet peaks at δ = 2.20 and δ = 2.06 ppm with *J* = 16 Hz and a singlet peak at δ = 2.95 ppm. Two sharp singlet peaks at δ = 1.13 and δ = 1.09 ppm are attributed to the 2CH_3_ groups in dimedone moiety.

### Spectral data

*6,6-Dimethyl-9-(2,4-dichloro)-2-phenyl-6,7-dihydro-[1,2,4]triazolo[1,2-a]indazole-1,3,8(2H,5H,9H)-trione(4a)*: white powder**;** IR (KBr,cm^-1^): 2960 (CH stretch), 1782, 1729, 1668 (C=O stretch), 1642,1566 (C=C aromatic stretch); ^1^H NMR (DMSO-d_6_, 400 MHz) δ (ppm): 7.67 (s, 1H, Ar–H), 7.53–7.48 (m, 7H, Ar–H**),** 6.38 (s, 1H, CH), 2.91–2.78 (m, 2H, CH_2_), 2.29–2.25 (d, *J* = 16 Hz, 1H, CH), 2.25–2.21 (d, *J* = 16 Hz, 1H, CH), 1.13 (s, 6H, 2 CH_3_). (See SI, Figs. S1, S2).

*6,6-Dimethyl-9-(4-nitrophenyl)-2-phenyl-6,7-dihydro-[1,2,4]triazolo[1,2-a]indazole-1,3,8 (2H,5H,9H)-trione (4b)*: white powder; IR (KBr,cm^-1^): 3177, 2951 (CH stretch), 1766, 1702 (C=O stretch), 1611 (C=C stretch); ^1^H NMR (DMSO, 400 MHz) δ (ppm): 8.20–8.18 (d, *J* = 8 Hz, 2H, Ar–H), 7.51–7.43 (m, 7H, Ar–H), 6.66 (s, 1H, CH), 2.92–2.78 (m, 2H, CH_2_), 2.39–2.35 (d, *J* = 16 Hz, 1H, CH_2_), 2.32–2.28 (d, *J* = 16 Hz, 1H, CH_2_), 1.04 (s, 6H, 2 CH_3_). (See SI, Figs. S3, S4).

*6,6-Dimethyl-2,9-diphenyl-6,7-dihydro-[1,2,4]triazolo[1,2-a]indazole-1,3,8(2H,5H,9H)-trione(4c)*: white powder; IR (KBr,cm^-1^): 3487(overtone), 2952 (C-H stretch), 1782, 1726, 1666 (C=O stretch), 1617 (C=C stretch); ^1^H NMR (DMSO, 400 MHz) δ (ppm); 7.54–7.43 (m, 6H, Ar–H), 7.35–7.33 (d, *J* = 8 Hz, 2H, Ar–H), 6.95–6.93 (d, *J* = 8 Hz, 2H, Ar–H), 6.00 (s, 1H, CH), 2.90–2.78 (m, 2H, CH_2_), 2.35–2.31 (d, *J* = 16 Hz, 1H, CH_2_), 2.20–2.16 (d, *J* = 16 Hz, 1H, CH_2_), 1.13 (s, 6H, 2CH_3_). (See SI, Figs. S5, S6).

*6,6-Dimethyl-9-(3-nitrophenyl)-2-phenyl-6,7-dihydro-[1,2,4]triazolo[1,2-a]indazole-1,3,8 (2H,5H,9H)-trione (4d)* IR (KBr,cm^-1^): white powder; 3086, 2961(C-H stretch), 1737 (C=O stretch),1597, 1527 (C = C stretch); ^1^H NMR (DMSO, 400 MHz) δ (ppm): 8.32 (s, 1H, Ar–H), 8.20 (d, *J* = 8.0 Hz,1H, Ar–H), 7.96 (d, *J* = 8.0 Hz, 1H, Ar–H), 7.71 (t, *J* = 8.0 Hz, 1H, Ar–H), 7.56–7.44 (m, 5H, Ar–H), 6.23 (s, 1H, CH), 2.92–2.78 (m, 2H, CH_2_), 2.34 (d, *J* = 16.0 Hz, 1H, CH_2_), 2.16 (d, *J* = 16.0 Hz, 1H, CH_2_), 1.07 (s, 3H, CH_3_), 1.03 (s, 3H, CH_3_). (See SI, Figs. S7, S8).

*6,6-Dimethyl-9-(4-chloro)-2-phenyl-6,7-dihydro-[1,2,4]triazolo[1,2-a]indazole-1,3,8 (2H,5H,9H)-trione (4e)*: white powder; IR (KBr,cm^-1^): 3447 (C=O), 2924, 2859 (C-H stretch), 1749 (C=C aromatic stretch), 1539 (C=C stretch); ^1^H NMR (DMSO-d6, 400 MHz δ (ppm): 8.27- 8.25 (d, *J* = 8 Hz, 2H, Ar–H), 7.69–7.67 (d, *J* = 8 Hz, 2H, Ar–H), 7.53- 7.48 (m, 5H, Ar–H), 6.30 (s, 1H, CH), 2.6 (s, 2H, CH_2_), 2.28–2.24 (d, *J* = 16 Hz, 1H, CH_2_), 2.19–2.15 (d, *J* = 16 Hz, 1H, CH_2_), 1.12 (s, 3H, CH_3_), 0.9 (s, 3H, CH_3_). (See SI, Figs. S9, S10).

*6,6-Dimethyl-2-phenyl-6,7-dihydro-1H-spiro[[1,2,4]triazolo[1,2-a]indazole-9,30-indoline]-1,20,3,8(2H,5H)-tetraone (4f.)*: orange powder; IR (KBr,cm^−1^): 3439 (N–H), 2957 (CH stretch), 1782, 1726, 1665 (C=O stretch), 1622 (C=C stretch); ^1^H NMR (DMSO-d6, 400 MHz) δ (ppm): 10.93 (s, NH), 7.53- 7.50 (m, 2H, Ar–H), 7.48- 7.42 (m, 4 Hz, Ar–H), 7.31.7.28 (t, *J* = 12 Hz, 1H, Ar–H),7.28–6.98 (t, *J* = 16 Hz, 1H, Ar–H), 6.91–6.89 (d, *J* = 8 Hz, 1H, Ar–H), 2.95 (s, 2H, CH_2_), 2.22–2.18 (d, *J* = 16 Hz, 1H, CH_2_), 2.08–2.04 (d, *J* = 16 Hz, 1H, CH_2_), 1.13 (s, 3H, CH_3_), 1.09 (s, 3H, CH_3_). (See SI, Figs. S11, S12).

*5'-Nitro-6,6-Dimethyl-2-phenyl-6,7-dihydro-1H-spiro[[1,2,4]triazolo[1,2-a]indazole-9,30-indoline]-1,20,3,8(2H,5H)-tetraone (4 g)*: yellow powder; IR (KBr,cm^-1^): 3214 (N–H stretch), 3069 (C-H stretch), 1737, 1674 (C=O stretch), 1620 (C=C stretch); ^1^H NMR (DMSO-d6, 400 MHz) δ (ppm): 10.46 (s, NH), 8.46–8.44 (d, *J* = 8 Hz, 1H, Ar–H), 8.23 (s, 1H, Ar–H), 7.53–7.39 (m, 5H, Ar–H), 7.10–7.08 (d, *J* = 8 Hz, 1H), 2.96–2.94 (m, 2H, CH_2_), 2.28–2.24 (d, *J* = 16 Hz, 1H, CH_2_), 2.19–2.15 (d, *J* = 16 Hz, 1H, CH_2_), 1.17 (s, 3H, CH_3_), 1.13 (s,3H, CH_3_). (See SI, Figs. S13, S14).

*5'-Bromo-6,6-dimethyl-2-phenyl-6,7-dihydro-1H-spiro[[1,2,4]triazolo[1,2-a]indazole-9,30-indoline]-1,20,3,8(2H,5H)-tetraone (4 h)*: white powder; IR (KBr,cm^-1^): 3410 (N–H stretch), 2958 (CH stretch), 1737 (C=O stretch), 1613 (C=C stretch); ^1^H NMR (DMSO-d6, 400 MHz) δ (ppm): 11.15 (s, 1H, N–H), 7.93 (m, 1H, Ar–H), 7.93–7.75 (d, *J* = 8.5, 1H, Ar–H), 7.73 (m, 1H), 7.63–7.45 (m, 4H, Ar–H), 6.89–6.87 (d, *J* = 8 Hz, 1H), 2.93 (m, 2H, CH_2_), 2.26 (s, 2H, CH_2_), 1.16 (s, 3H, CH_3_), 1.12 (s, 3H, CH_3_). (See SI, Figs. S15, S16).

#### Conclusion

In the literature, we first synthesized GO/f-SiO_2_/Co as a superb, efficient catalyst with high stability and high efficiency which was applied to the synthesis of triazolo[1,2-a]indazole-trione and spiro triazolo[1,2-a]indazole-tetraones under solvent-free conditions at 90 °C. The catalyst was prepared via conjugation of hybrid silane group with graphene oxide surface and chelation with Co(II). The catalyst showed high catalytic activity in the synthesis of target compounds and caused the first materials to change to the final products with high yields (> 93%). No difficulty in separation, resistance and reusability are some advantages of the catalyst. In addition, easy work method as well as high yields and short reaction times are favorable results of using the catalyst.

## Supplementary Information

Supplementary Information.

## Data Availability

All data generated or analyzed during this study are included in this published article and its supplementary information file.
