# Supplementary material for: Three component synthesis of triazolo[1,2-a]indazole-trione and spiro triazolo[1,2-a]indazole-tetraones using GO/SiO2/Co (II)
Source: Sci Rep. 2022 Oct 25;12:17830. doi: 10.1038/s41598-022-22304-y (PMC9596706; doi:10.1038/s41598-022-22304-y)
Supplement: Supplementary file 1 — Supplementary Information. [file 41598_2022_22304_MOESM1_ESM.docx]

**Three component synthesis of** **triazolo[1,2-a]indazole-trione and spiro triazolo[1,2-a]indazole-tetraones using GO/SiO_2_/Co (II)**

Mahnaz Mirheidari^1^, Javad Safaei-Ghomi*^1^

^1^Department of Organic Chemistry, Faculty of Chemistry, University of Kashan, Kashan, I. R. Iran,

*E-mail address: [safaei@kashanu.ac.ir](mailto:safaei@kashanu.ac.ir).


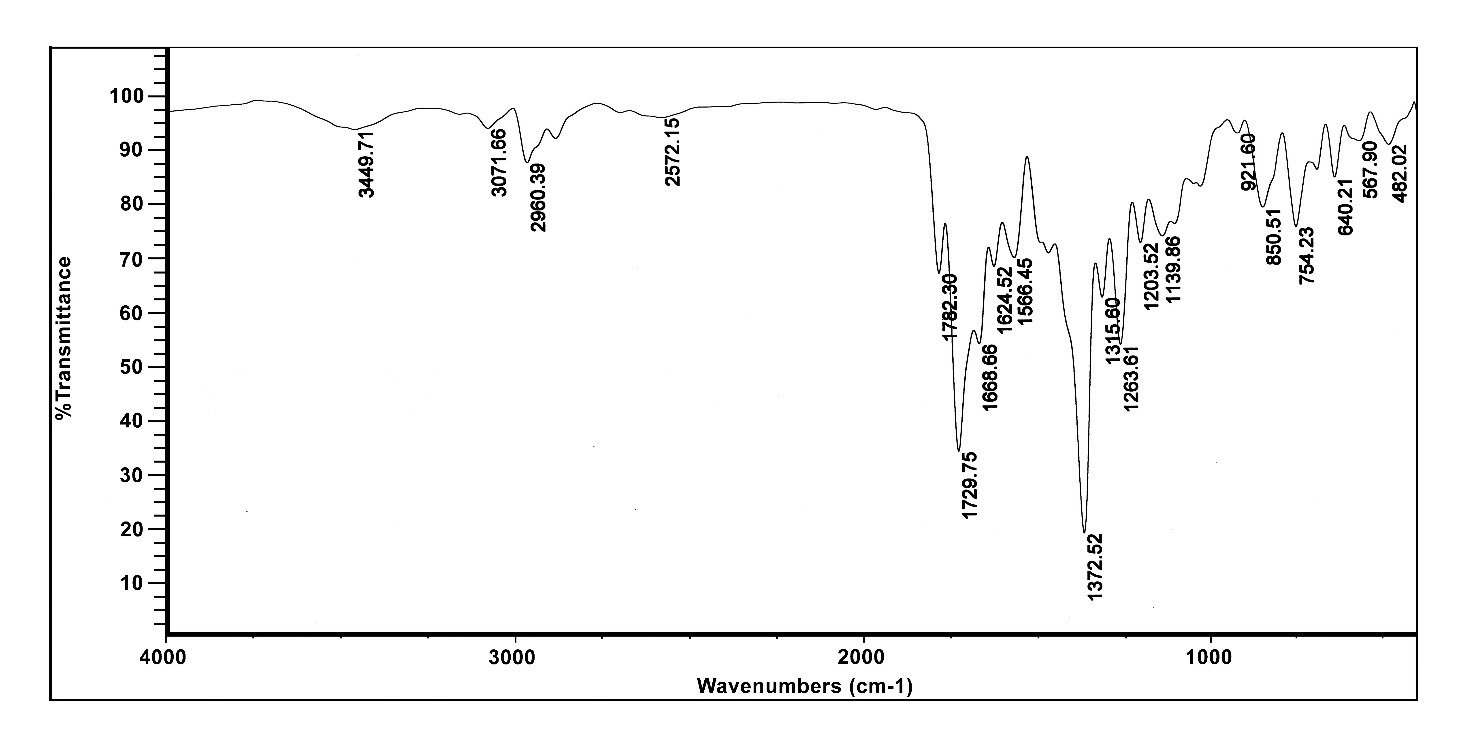


Figure S1: FT-IR spectrum of compound 4a


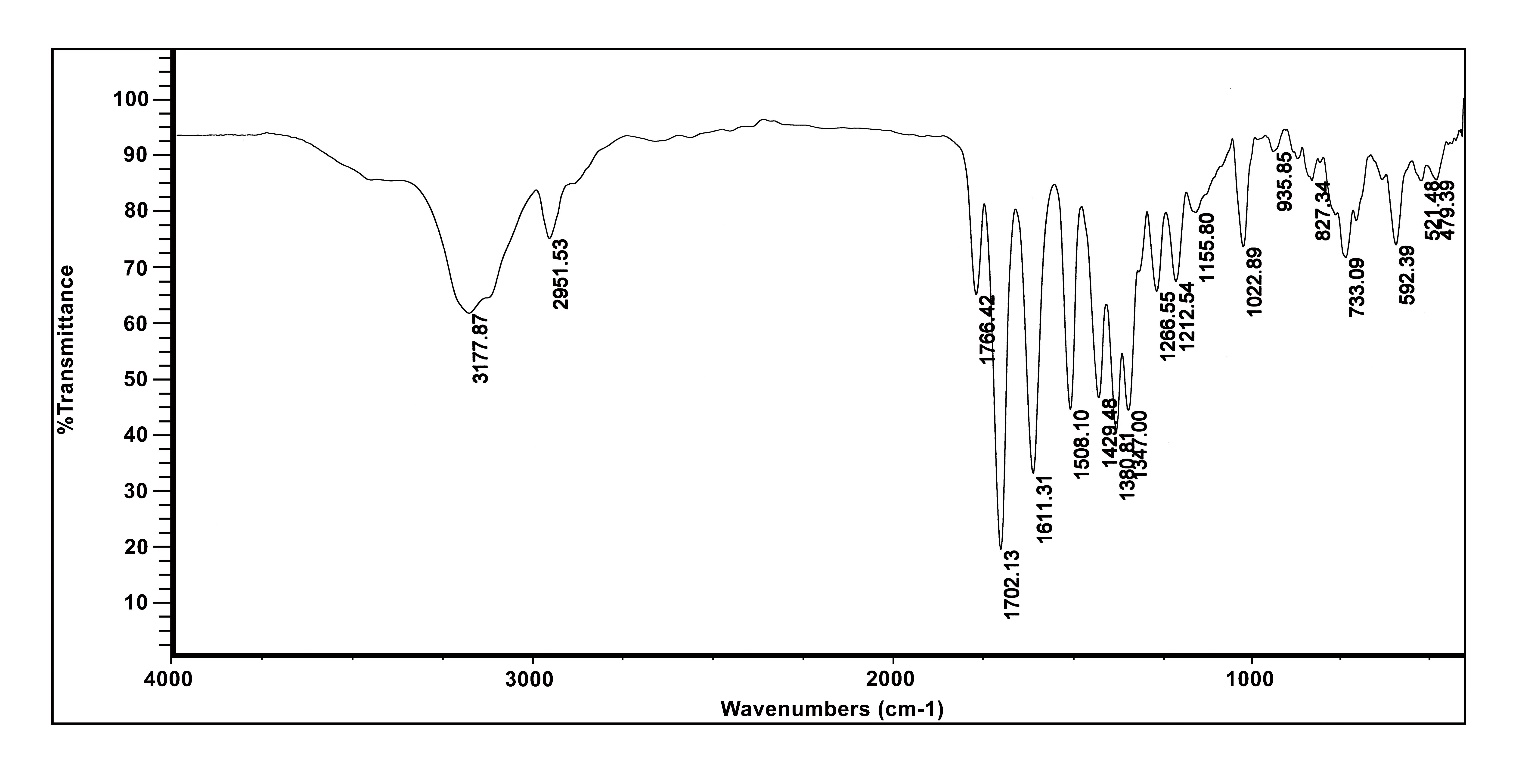


Figure S3: FT-IR spectrum of compound 4b

Figure S2: ^1^H NMR spectrum of compound 4a


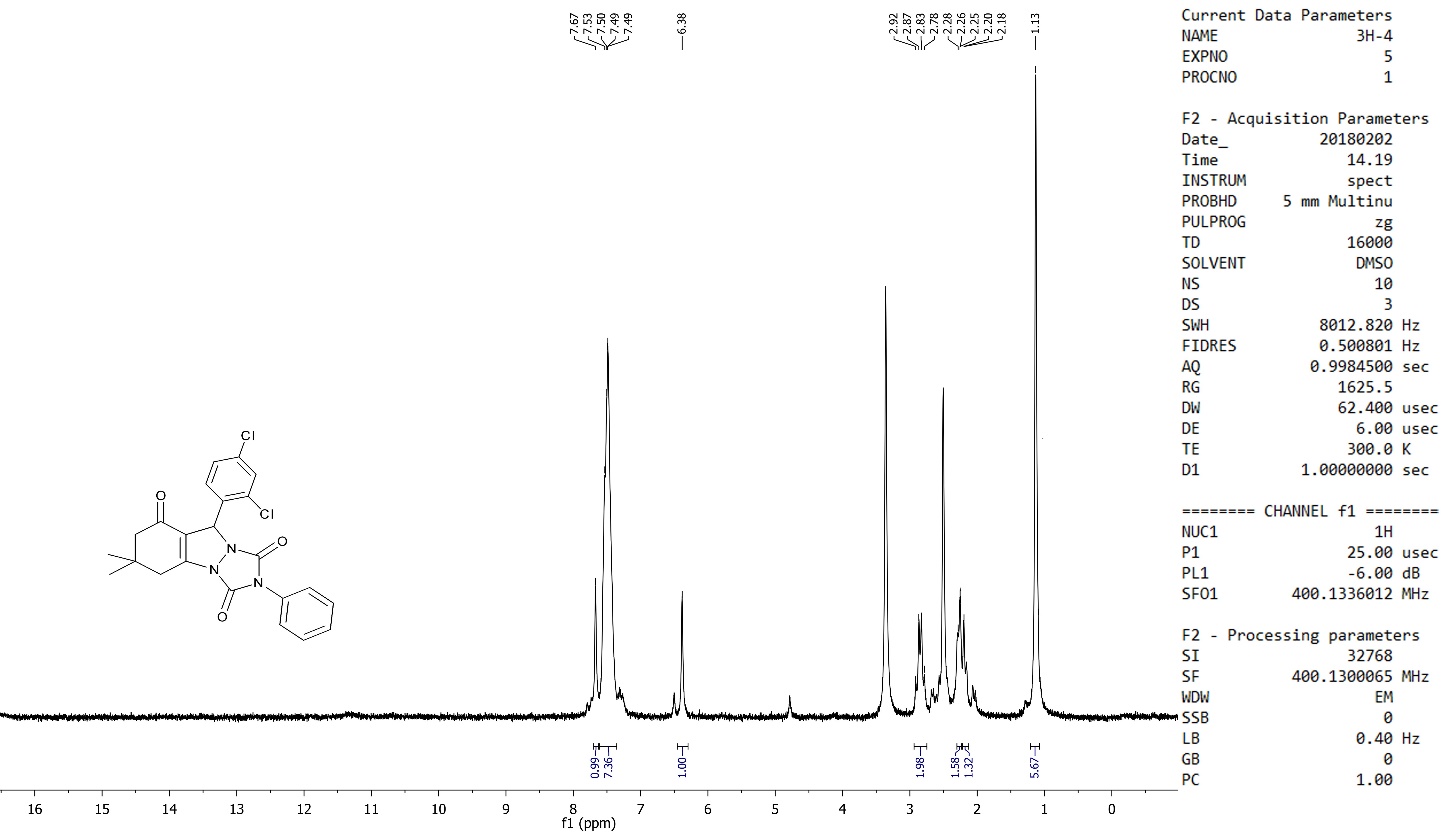


Figure S4: ^1^H NMR spectrum of compound 4b


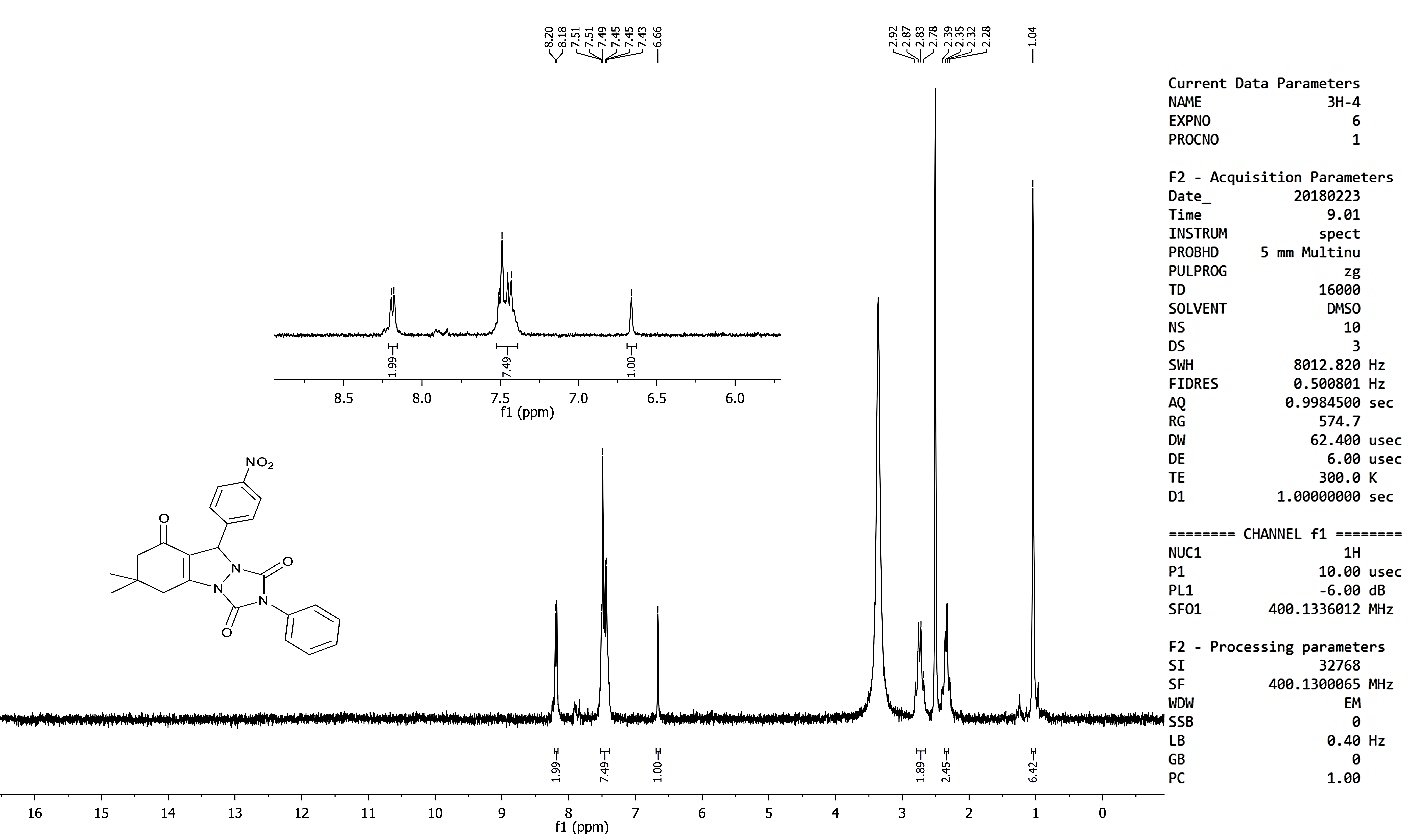

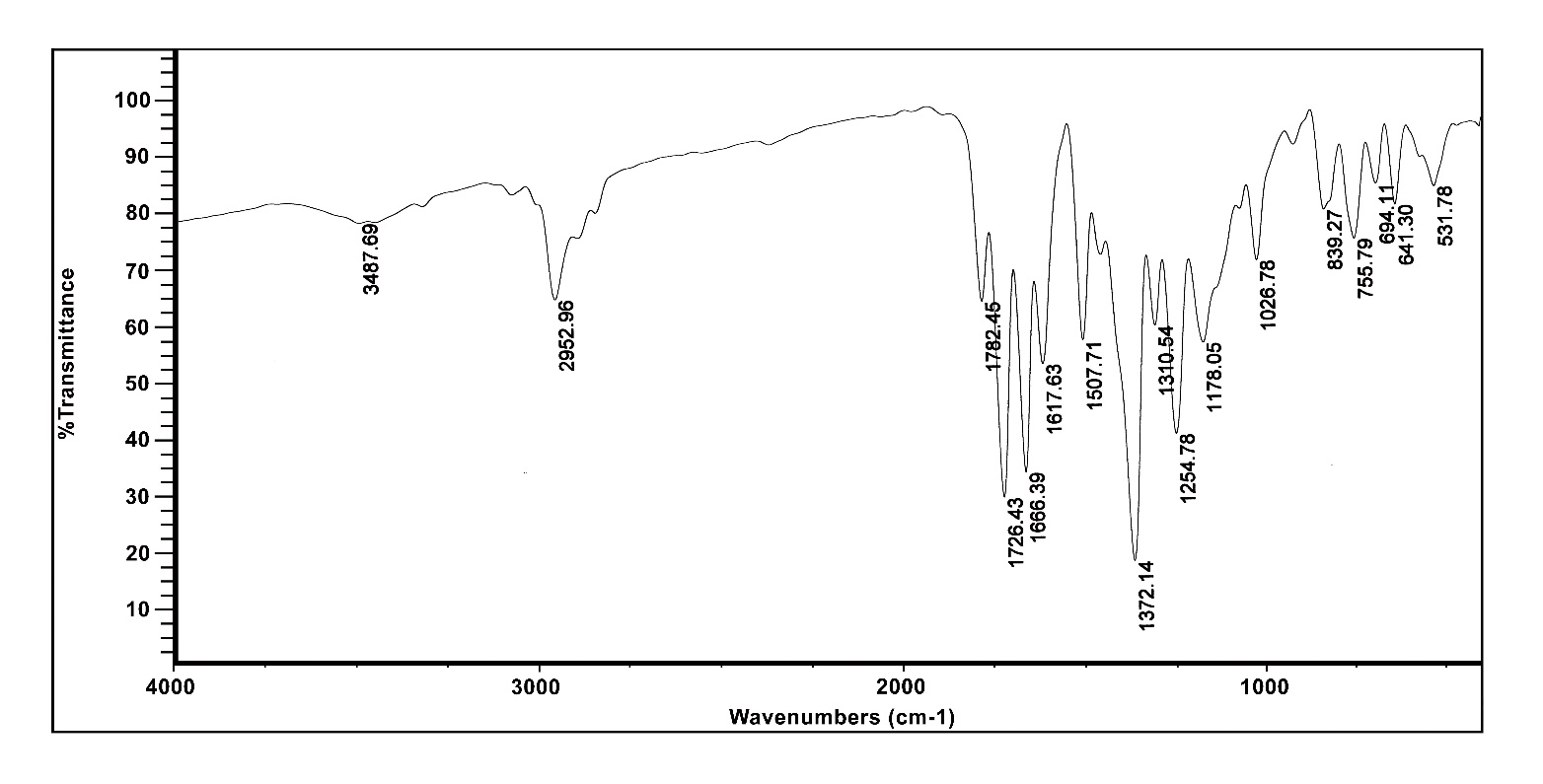


Figure S5: FT-IR spectrum of compound 4c


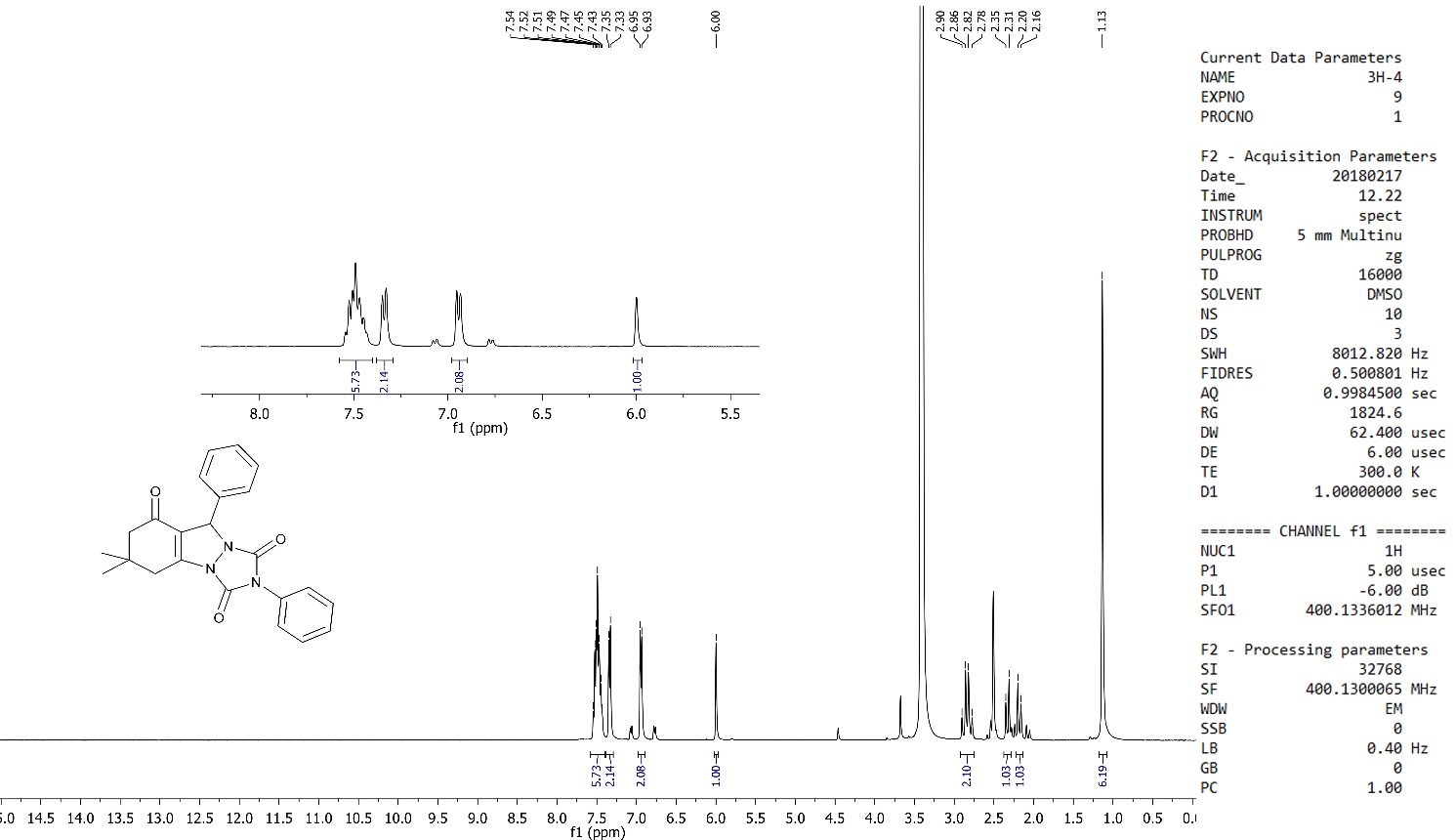


Figure S6: ^1^H NMR spectrum of compound 4c


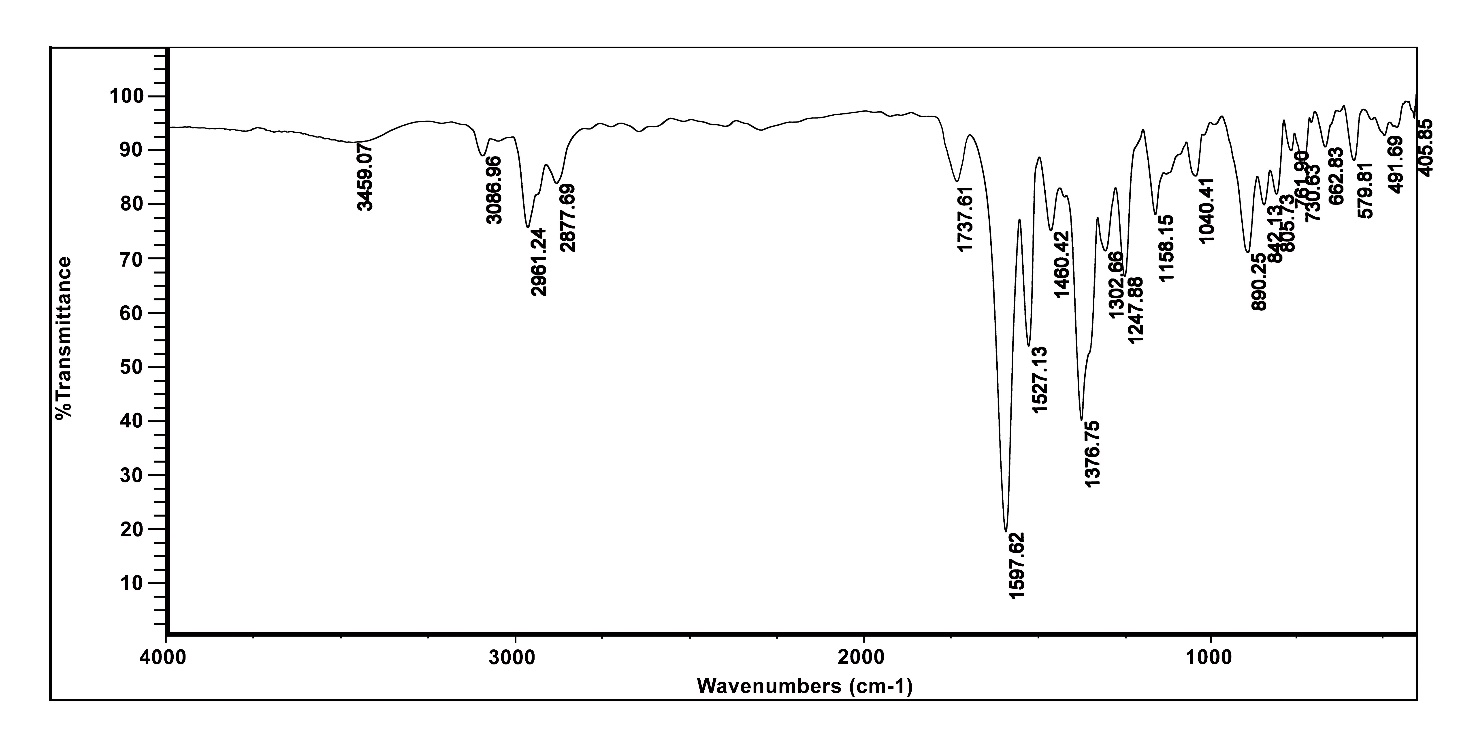


Figure S7: FT-IR spectrum of compound 4d


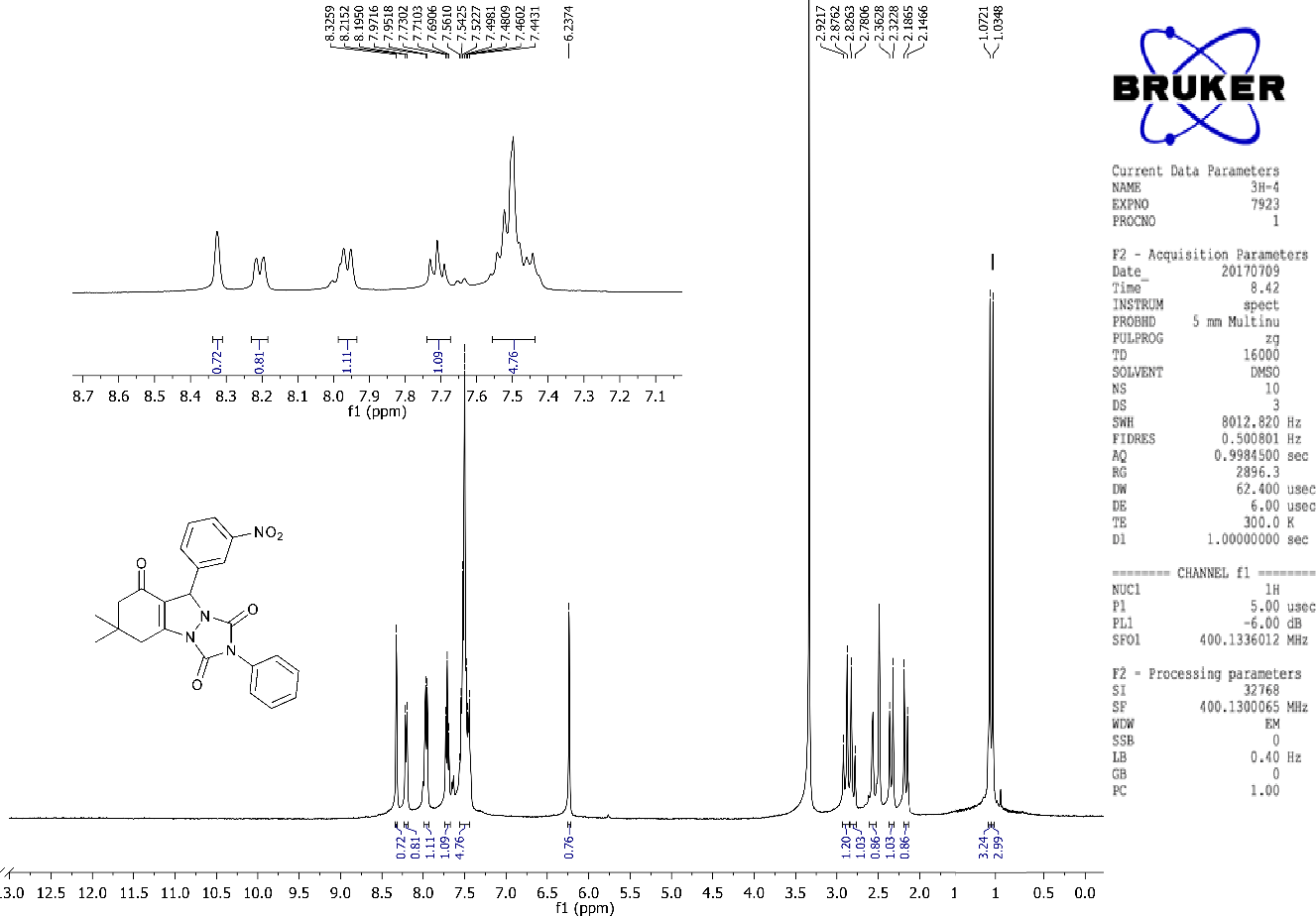


Figure S8: ^1^H NMR spectrum of compound 4d


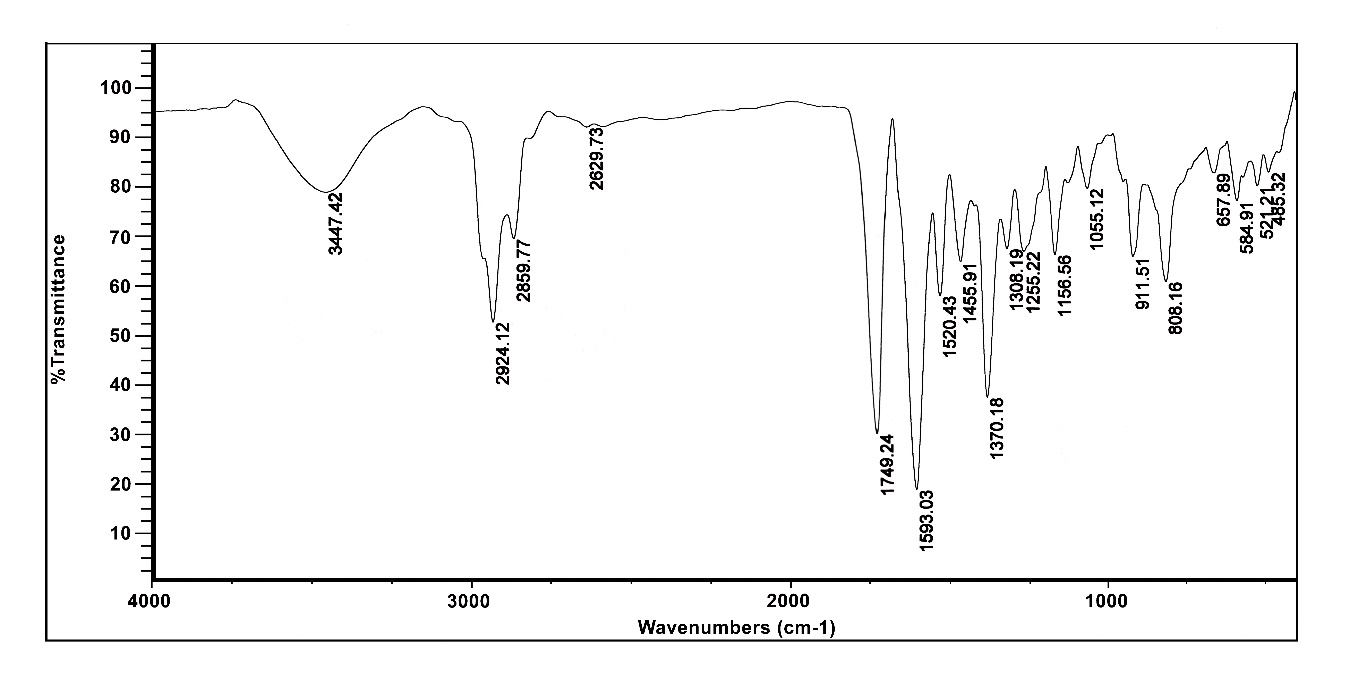


Figure S9: FT-IR spectrum of compound 4e


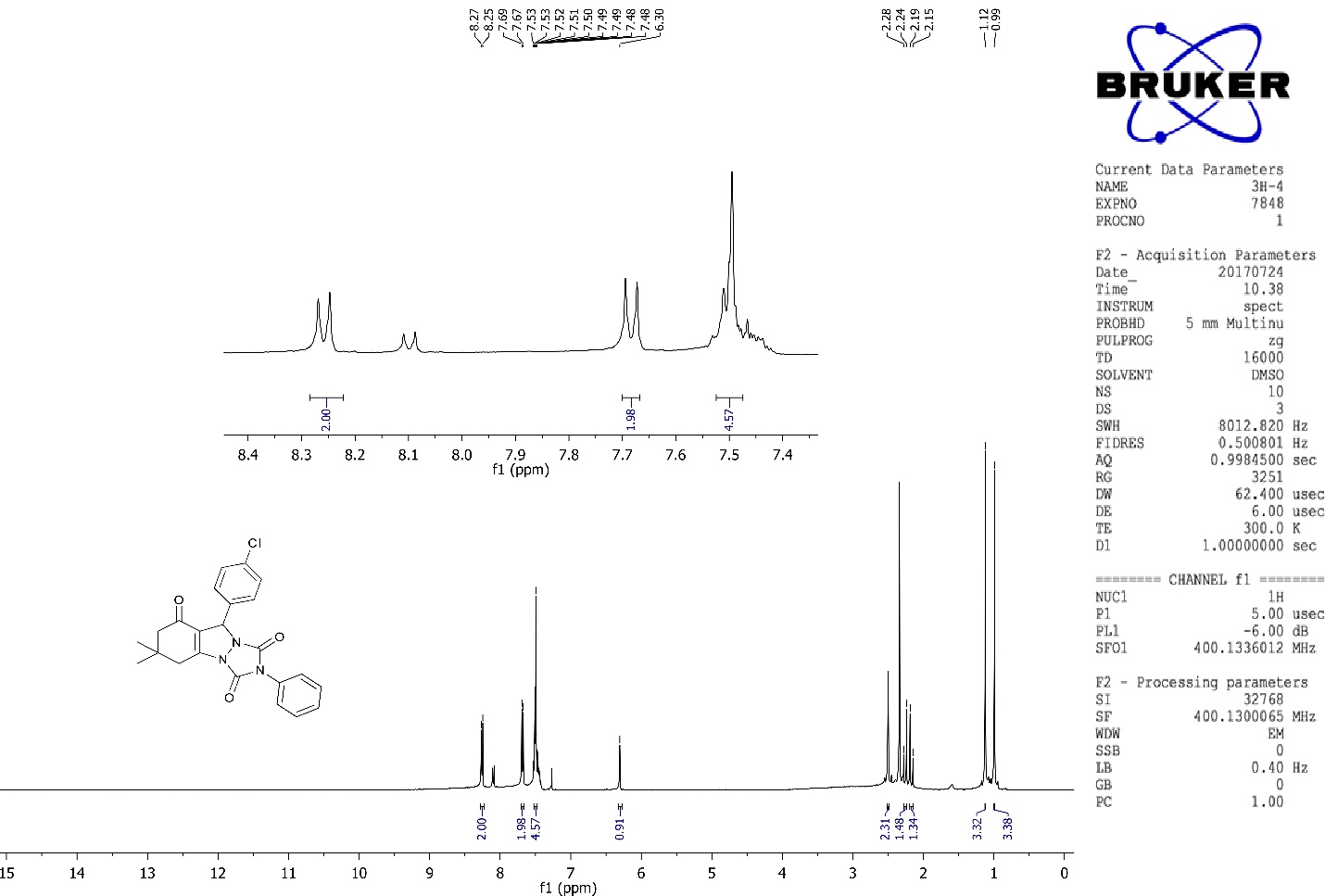


Figure S10: ^1^H NMR spectrum of compound 4e


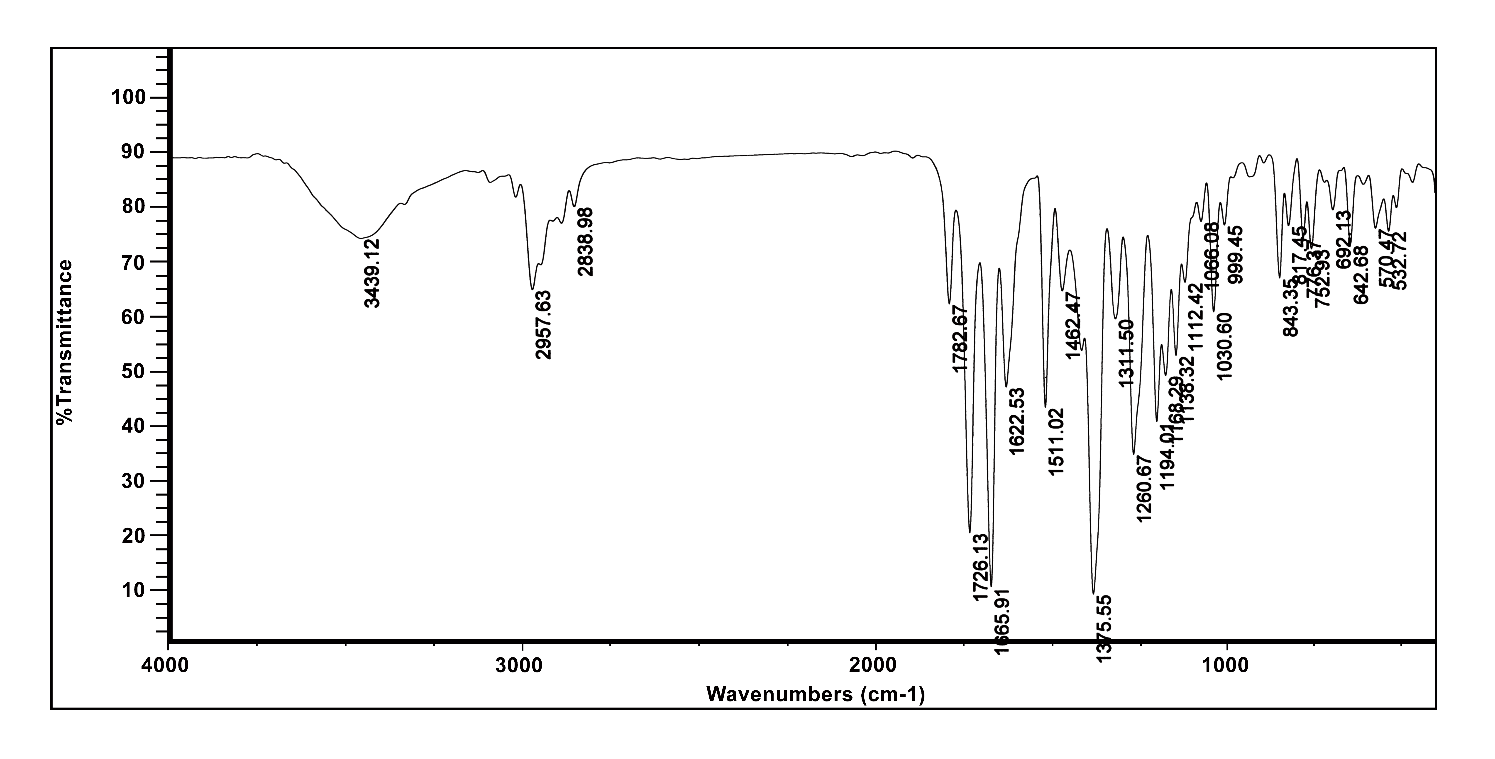


Figure S11: FT-IR spectrum of compound 4f


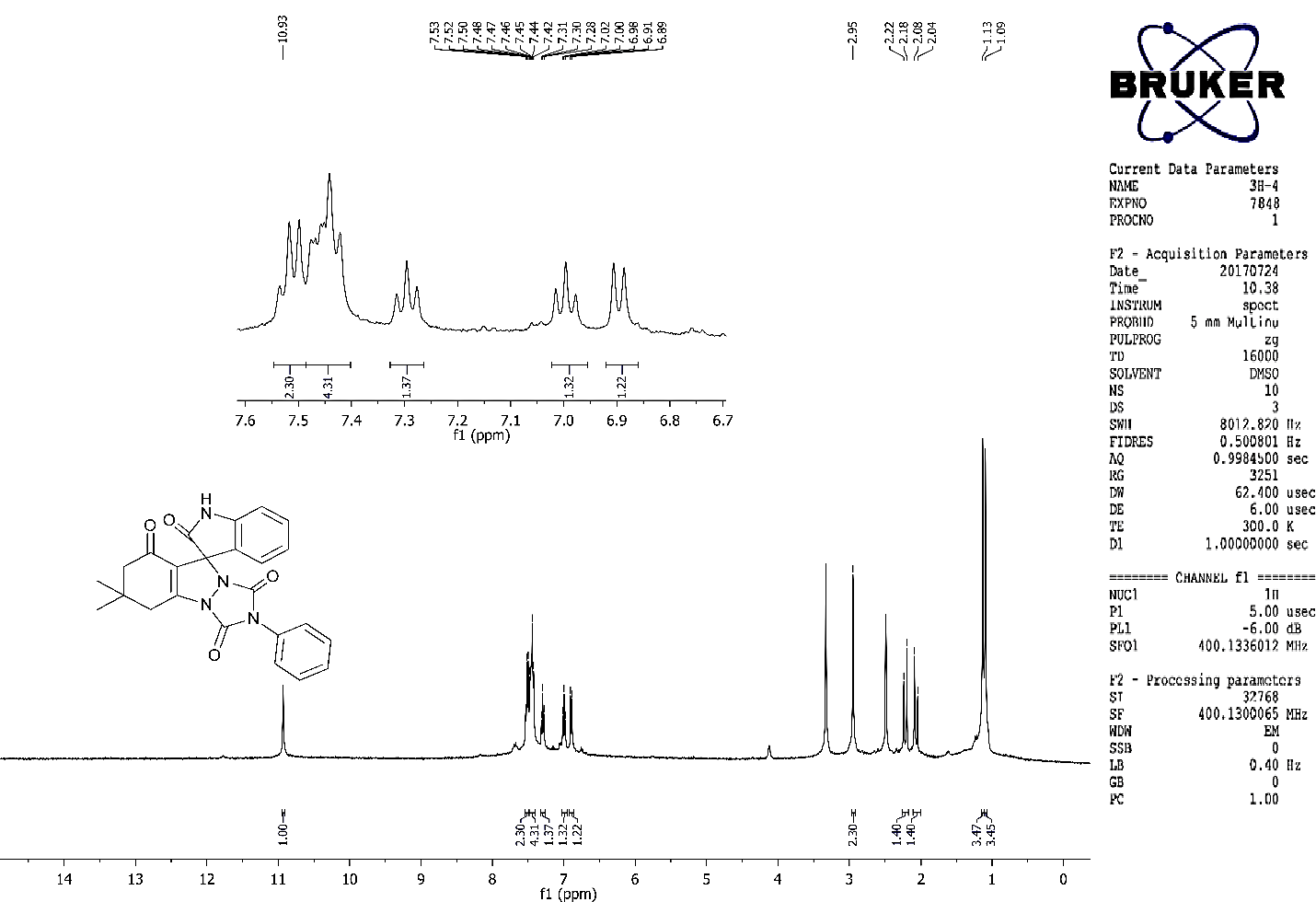


Figure S12: ^1^H NMR spectrum of compound 4f


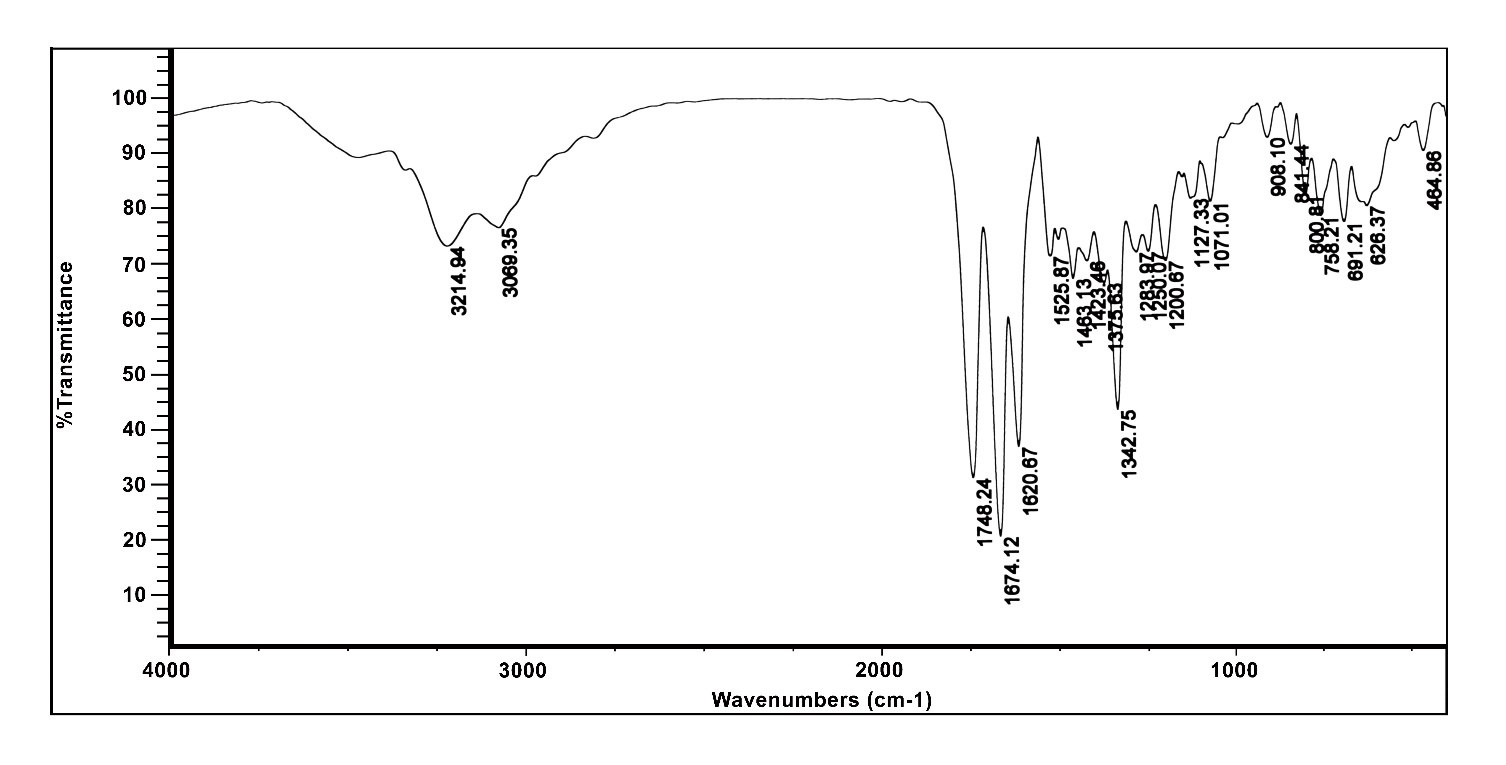


Figure S13: FT-IR spectrum of compound 4g


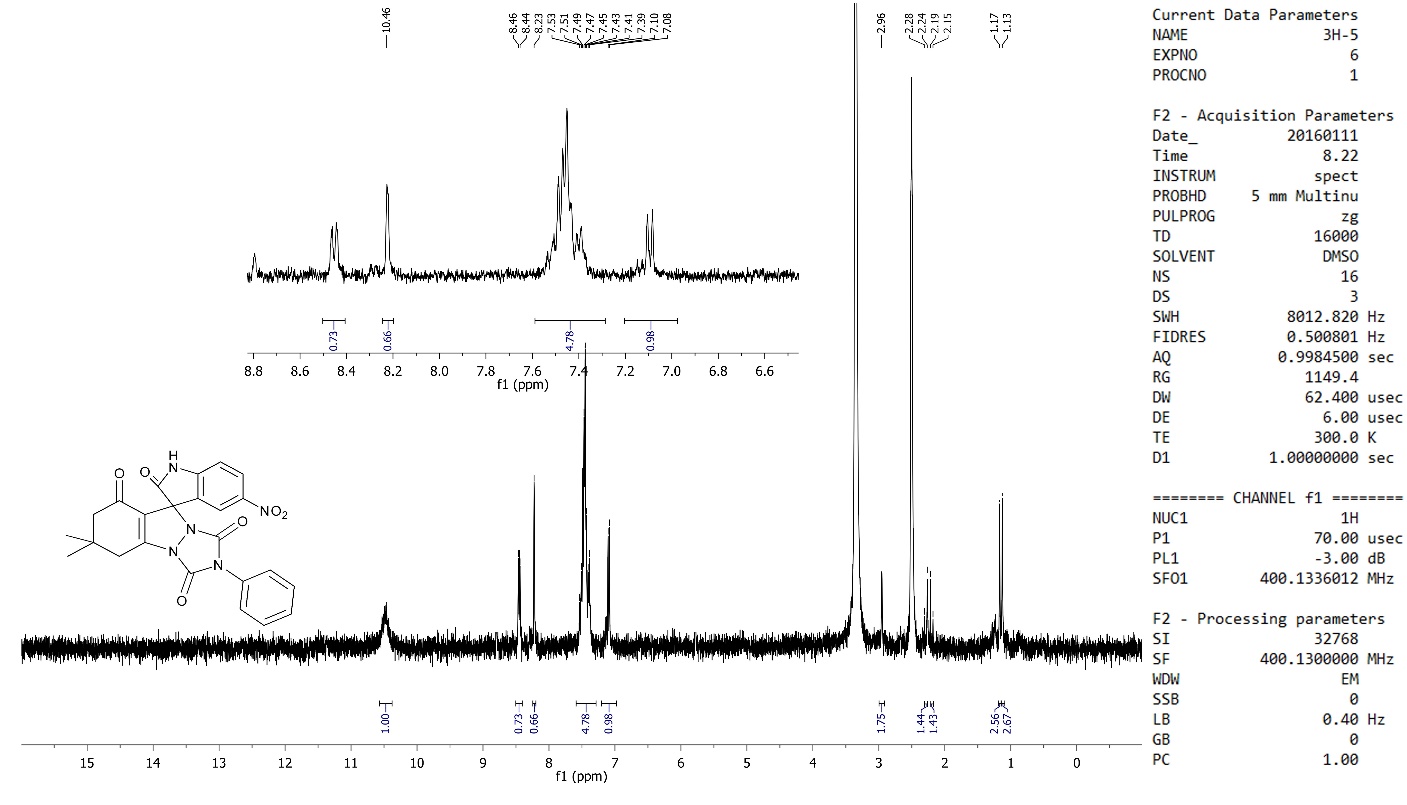


Figure S14: ^1^H NMR spectrum of compound 4g


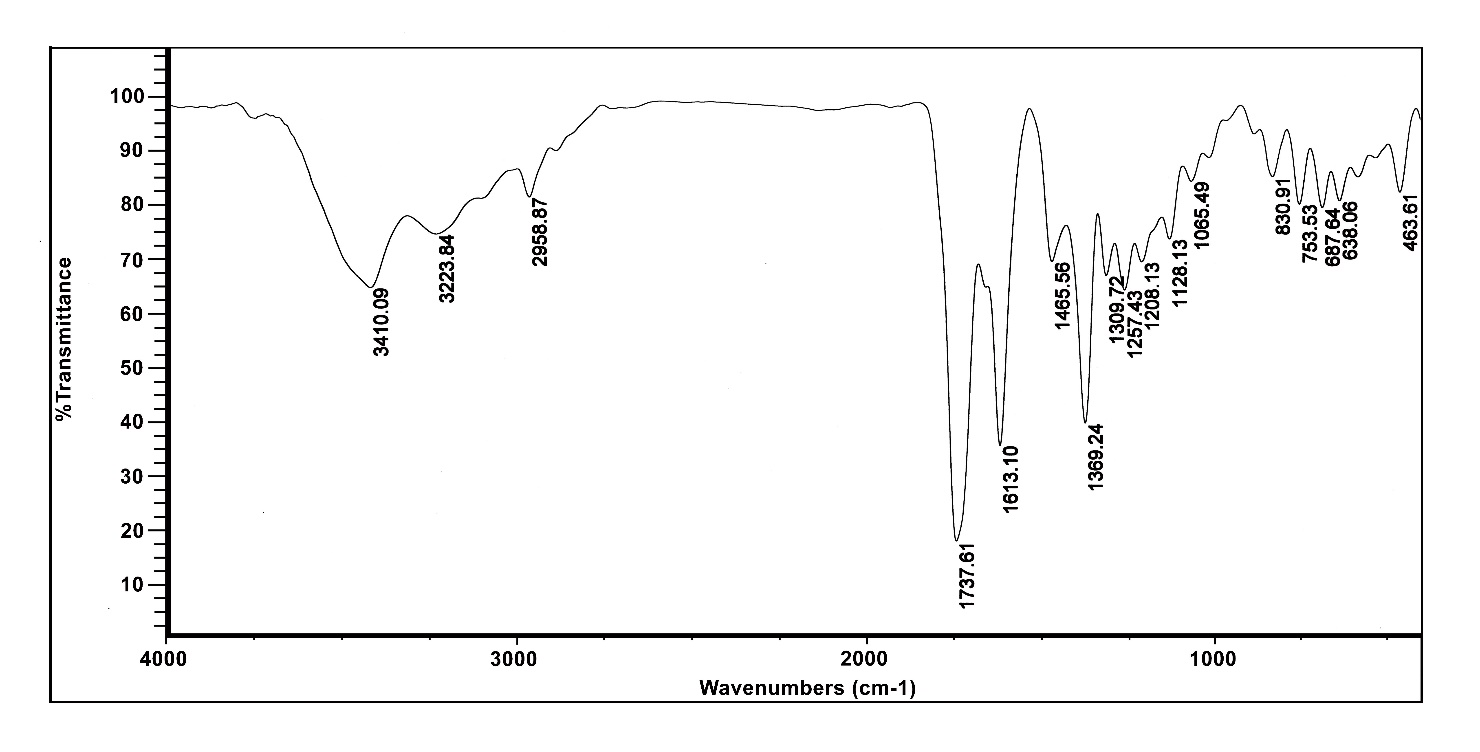


Figure S15: FT-IR spectrum of compound 4h


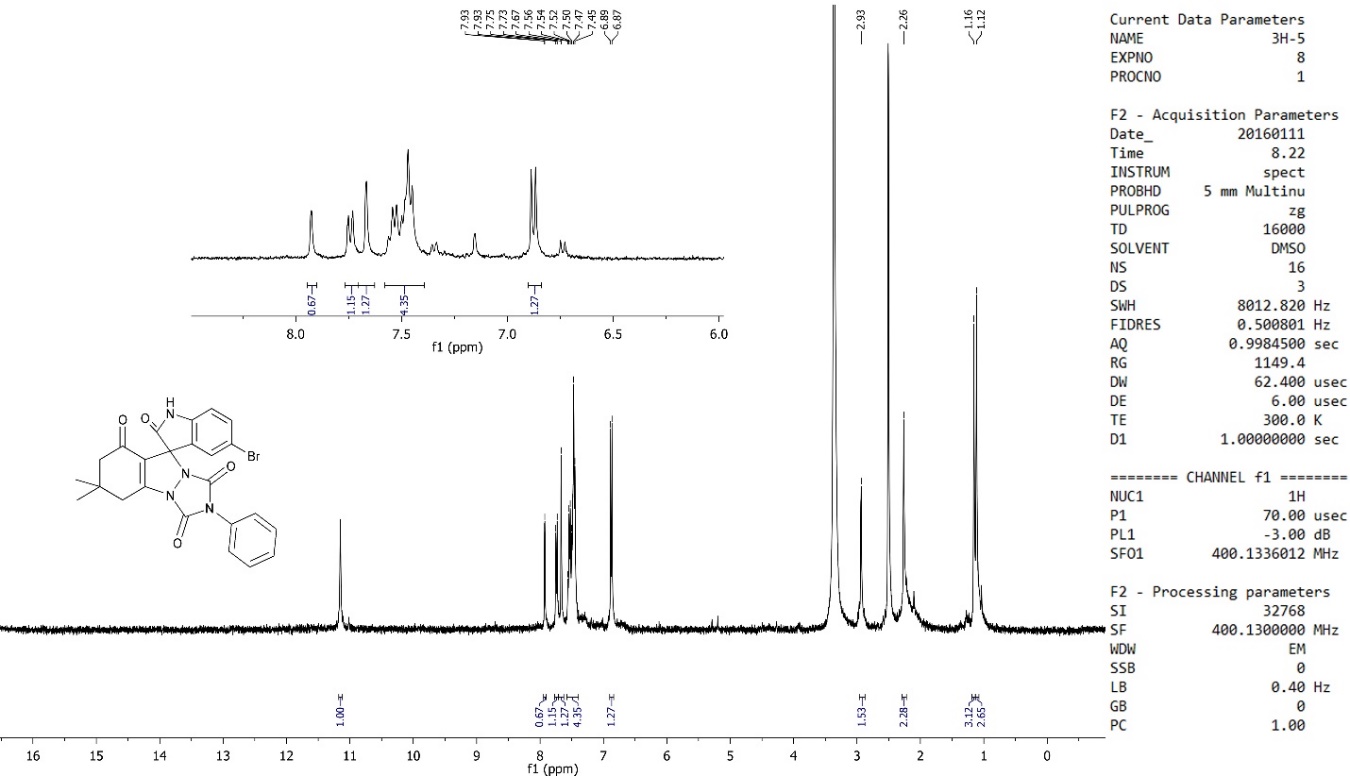


Figure S16: ^1^H NMR spectrum of compound 4h
